# Facile Affinity Maturation of Antibody Variable Domains Using Natural Diversity Mutagenesis

**DOI:** 10.3389/fimmu.2017.00986

**Published:** 2017-09-04

**Authors:** Kathryn E. Tiller, Ratul Chowdhury, Tong Li, Seth D. Ludwig, Sabyasachi Sen, Costas D. Maranas, Peter M. Tessier

**Affiliations:** ^1^Isermann Department of Chemical and Biological Engineering, Center for Biotechnology and Interdisciplinary Studies, Rensselaer Polytechnic Institute, Troy, NY, United States; ^2^Department of Chemical Engineering, The Pennsylvania State University, University Park, PA, United States

**Keywords:** complementarity-determining region, stability, specificity, library, directed evolution, yeast surface display, protein design

## Abstract

The identification of mutations that enhance antibody affinity while maintaining high antibody specificity and stability is a time-consuming and laborious process. Here, we report an efficient methodology for systematically and rapidly enhancing the affinity of antibody variable domains while maximizing specificity and stability using novel synthetic antibody libraries. Our approach first uses computational and experimental alanine scanning mutagenesis to identify sites in the complementarity-determining regions (CDRs) that are permissive to mutagenesis while maintaining antigen binding. Next, we mutagenize the most permissive CDR positions using degenerate codons to encode wild-type residues and a small number of the most frequently occurring residues at each CDR position based on natural antibody diversity. This mutagenesis approach results in antibody libraries with variants that have a wide range of numbers of CDR mutations, including antibody domains with single mutations and others with tens of mutations. Finally, we sort the modest size libraries (~10 million variants) displayed on the surface of yeast to identify CDR mutations with the greatest increases in affinity. Importantly, we find that single-domain (V_H_H) antibodies specific for the α-synuclein protein (whose aggregation is associated with Parkinson’s disease) with the greatest gains in affinity (>5-fold) have several (four to six) CDR mutations. This finding highlights the importance of sampling combinations of CDR mutations during the first step of affinity maturation to maximize the efficiency of the process. Interestingly, we find that some natural diversity mutations simultaneously enhance all three key antibody properties (affinity, specificity, and stability) while other mutations enhance some of these properties (e.g., increased specificity) and display trade-offs in others (e.g., reduced affinity and/or stability). Computational modeling reveals that improvements in affinity are generally not due to direct interactions involving CDR mutations but rather due to indirect effects that enhance existing interactions and/or promote new interactions between the antigen and wild-type CDR residues. We expect that natural diversity mutagenesis will be useful for efficient affinity maturation of a wide range of antibody fragments and full-length antibodies.

## Introduction

The widespread interest in using antibodies in diagnostic and therapeutic applications has led to considerable efforts in developing methods for optimizing their properties (1–6). Methods for improving antibody affinity are particularly important because lead antibodies identified using *in vivo* (immunization) and *in vitro* (e.g., phage display) methods typically do not have high enough affinity for therapeutic applications. Moreover, improvements in antibody affinity are generally expected to enhance the performance of diagnostic antibodies due to improved specificity at reduced antibody concentrations. Methods such as phage, yeast surface and ribosome display are commonly used for *in vitro* affinity maturation because of their many attractive properties (7–13). These properties include the ability to precisely control antigen presentation, conformation, and concentration as well as the ability to perform negative selections against various types of non-antigens to eliminate non-specific variants (14–17). These display methods have been used to achieve large enhancements in affinity for a wide variety of antibody fragments and full-length antibodies (9, 18–23).

Nevertheless, there are several outstanding challenges related to *in vitro* affinity maturation that need to be addressed. First, while it is possible to use saturation mutagenesis to evaluate every possible single mutation in antibody complementarity-determining regions (CDRs), single mutations typically do not result in large gains in affinity (1, 3, 24). Therefore, it is often necessary to generate sub-libraries to identify combinations of single mutations that result in large increases in affinity, which is a slow and laborious process. Second, it is not possible to test all combinations of single and multiple mutations in the CDRs of antibodies in a single library due to intractably large library sizes. For example, a library size of >10^39^ would be required to sample all possible combinations of single and multiple mutations at ~30 residues in the CDRs of typical variable domains. This means that only an extremely small subset of the possible single and multiple mutations can be tested using display methods, which is largely dictated by transformation efficiencies [~10^9^–10^10^ for phage (25, 26) and ~10^7^–10^8^ for yeast (9, 27) using conventional transformation methods]. Therefore, it is important to develop smart library design methods that sample a relatively small number of residues at each CDR position that are most likely to generate antibodies with significant gains in affinity (28–41).

A third common challenge related to antibody affinity maturation is the identification of affinity-enhancing mutations that lead to reductions in antibody specificity (42–44). Highly interactive residues—such as arginine and aromatic residues—can be readily enriched in the CDRs during affinity maturation, which is concerning because they have increased risk for promoting non-specific interactions (43–47). While negative selections are useful for removing some non-specific variants, it is critical to use libraries with the highest possible fraction of specific variants to maximize the likelihood of isolating antibodies with not only increased affinity but also with high specificity. A related problem is that affinity-enhancing CDR mutations can lead to reductions in stability (48–51). Antibody affinity/stability trade-offs appear to be due to structural changes in the CDRs and frameworks that are necessary to increase affinity, and additional compensatory mutations are needed in some cases to maintain thermodynamic stability (48, 49, 51). Therefore, it is important to generate antibody libraries with the highest possible fraction of stable antibodies to minimize the frequency of isolating destabilized antibodies that require additional mutagenesis to restore stability.

**Figure 1:**
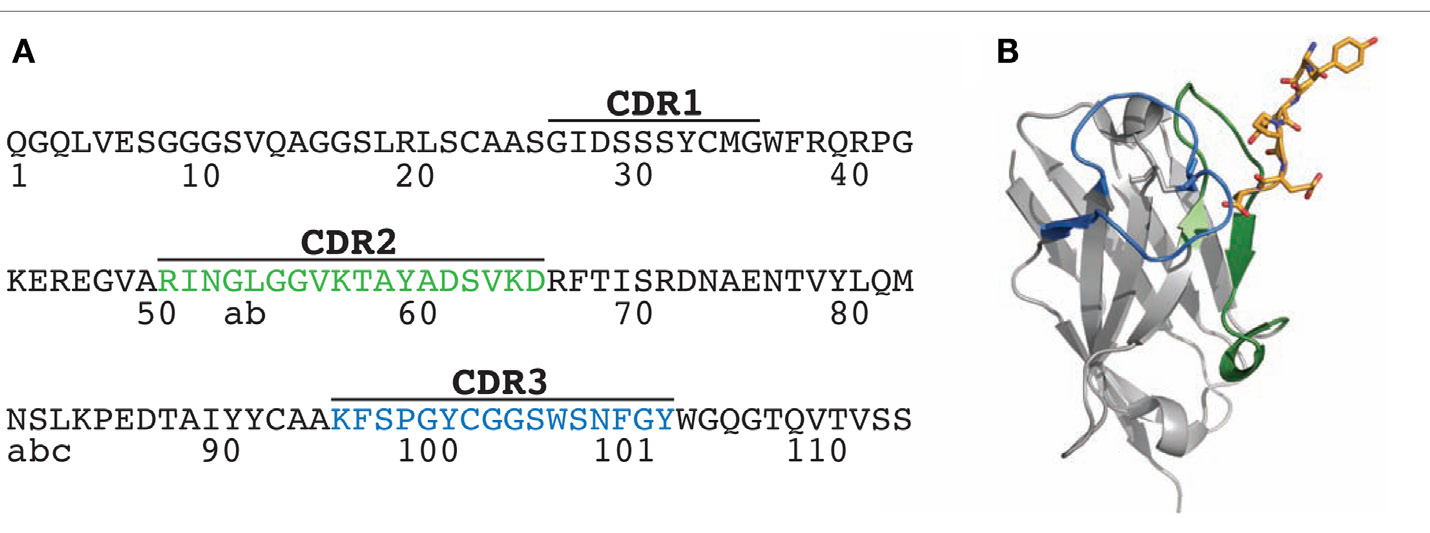
Sequence and structure of the N2 V_H_H antibody. **(A)** Amino acid sequence of wild-type N2 V_H_H antibody (originally referred to as NbSyn2). The framework and complementarity-determining region (CDR) sequences are defined according to Kabat. **(B)** Structure of N2 in complex with its antigen, a C-terminal α-synuclein peptide (residues 132-GYQDYEPEA-140; PDB 2X6M). Two of the key N2 CDRs involved in antigen binding are highlighted in green (CDR2) and blue (CDR3), while the antigen (α-synuclein peptide) is highlighted in yellow stick form.

To evaluate potential solutions to these challenges, we have sought to identify mutations that increase the affinity of a camelid single-domain antibody specific for the C-terminus of α-synuclein (52) (Figure 1). This variable (V_H_H) domain—originally referred to as NbSyn2 and herein referred to as N2—was previously isolated from an immune library. We selected this antibody domain for further optimization because its crystal structure is available in complex with antigen at high resolution (Figure 1), it is relatively simple to display on the surface of yeast for *in vitro* selections relative to more complex multidomain (scFv) and/or multichain (Fab or IgG) antibodies, it has intermediate affinity (*K_D_* of 58 ± 9 nM) that can be further increased, and it has relatively high stability (apparent melting temperature of 68 ± 0.3°C). We posit that efficient affinity maturation of antibody variable domains such as N2 can be accomplished in three steps: (i) identification of the most permissive sites in the CDRs that can be mutated without large (negative) impacts on affinity using alanine scanning mutagenesis; (ii) sampling of a small number of mutations at each permissive CDR site that correspond to either the wild-type residue or residues most commonly observed in natural antibodies at each CDR site; and (iii) screening of all possible combinations of single and multiple natural diversity antibody mutations in a single library. Here, we test this methodology by identifying the most permissive CDR sites in N2 and use these findings to generate a single library that is based on natural antibody diversity and includes both single and multiple (up to 14) CDR mutations. We demonstrate how this library design approach can be used along with yeast surface display to identify stable and specific variable domains with increased affinity.

## Results

### Alanine Scanning Mutagenesis Reveals Permissive CDR Sites

Toward our goal of developing systematic and robust affinity maturation methods, we first sought to identify permissive sites in the CDRs of N2 that weakly impact antibody affinity using both computational and experimental methods. Two of the CDRs (CDR2 and CDR3) are involved in mediating antigen binding (Figure 1). Our computational alanine scanning analysis of these CDRs identified two residues in CDR2 (N52 and K56) and two residues in CDR3 (Y100 and W100e) that are sensitive to mutation (Table S1 in Supplementary Material). We tested these observations using experimental alanine scanning mutagenesis at 18 sites in CDR2 and CDR3. Three sites in these CDRs (R50, P98, and C100a) were excluded from this analysis because they were either shown previously to be involved in mediating antigen binding (52) or suspected to be important for antibody structure and stability.

**Figure 2:**
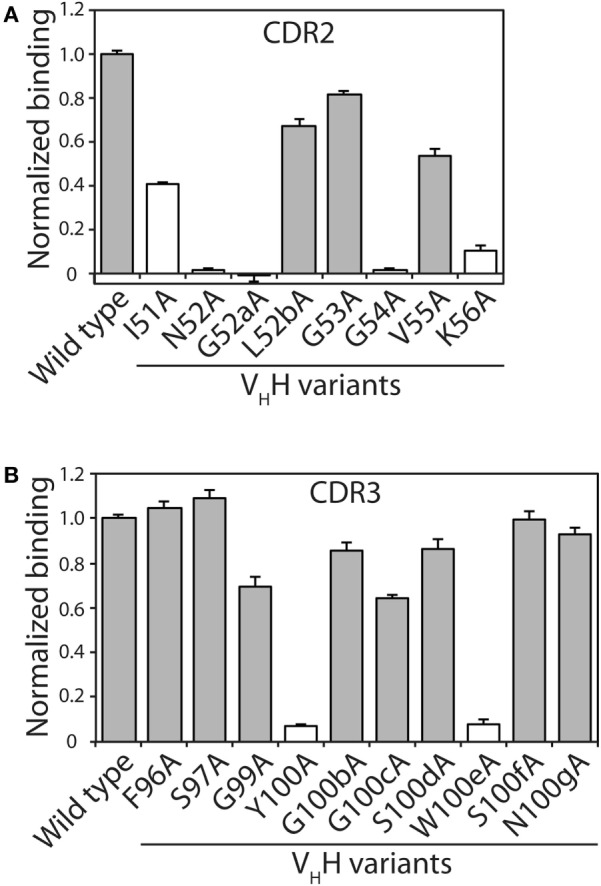
Identification of V_H_H complementarity-determining region (CDR) residues involved in antigen binding *via* alanine scanning mutagenesis. The relative antigen binding of the V_H_H variants (400 nM) with single alanine substitution mutations in **(A)** CDR2 and **(B)** CDR3 was evaluated using fluorescence polarization (2 nM TAMRA-labeled α-synuclein peptide). Raw polarization signals were background subtracted (background signals were obtained using samples with only TAMRA α-synuclein peptide), and normalized signals are reported (signal for mutant divided by that for wild type). Error bars represent the SD for three independent experiments. The V_H_H sequence is defined using Kabat numbering. Alanine mutants that have modest impacts on antigen binding (mutant binding is at least 50% of wild-type binding) are highlighted in gray fill, while those mutants with larger negative impacts on antigen binding are indicated in white fill.

The alanine mutants were expressed in bacteria and purified using metal-affinity chromatography (purification yields of 0.7–2.6 mg/L). SDS-PAGE analysis revealed high purities (Figure S1 in Supplementary Material). The relative binding of each mutant was evaluated using fluorescence polarization at three V_H_H concentrations (44, 133, and 400 nM; Figure 2; Figure S2 in Supplementary Material). Consistent trends were observed at each V_H_H concentration. Eleven of the 18 mutants retained >50% of the wild-type binding activity, including three in CDR2 (L52b, G53, and V55) and eight in CDR3 (F96, S97, G99, G100b, G100c, S100d, S100f, and N100g). The other seven mutants that displayed greater reductions in binding included five CDR2 mutants (I51, N52, G52a, G54, and K56) and two CDR3 mutants (Y100 and W100e), which were not subjected to further mutagenesis. Four of the disruptive mutations (N52 and K56 in CDR2 and Y100 and W100e in CDR3) were identified in our computational alanine scanning mutagenesis (Table S1 in Supplementary Material). These and other previous results (39, 53, 54) highlight the value of alanine scanning mutagenesis to identify permissive CDR sites that can be mutated during antibody affinity maturation.

### Design of Antibody Libraries Using Natural Diversity Mutagenesis

**Figure 3:**
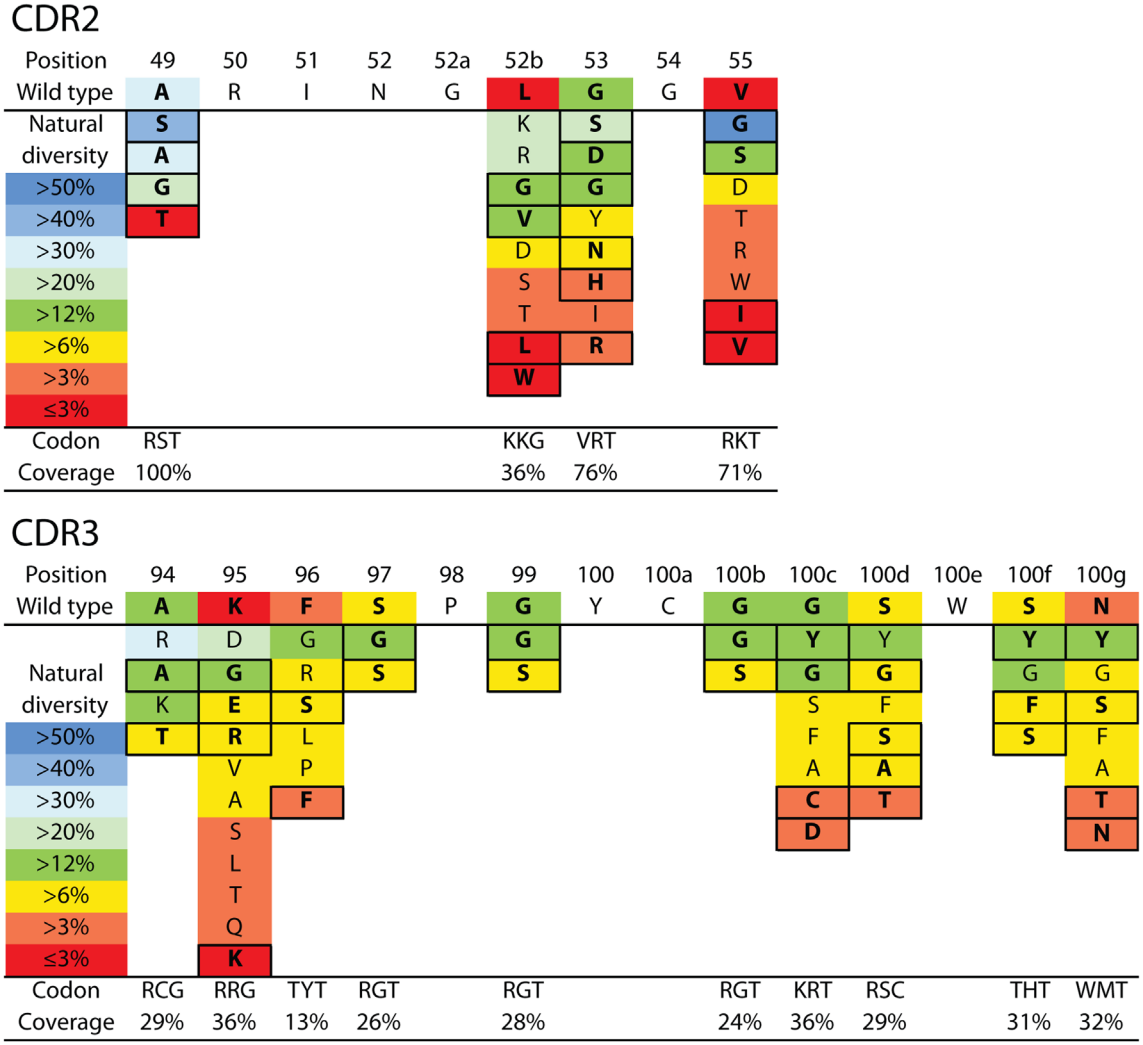
V_H_H library design for N2 affinity maturation using natural diversity mutagenesis. A single V_H_H library was designed that involved mutating four sites in CDR2 (top) and 10 sites in CDR3 (bottom). The CDR sites selected for mutagenesis were identified primarily using alanine scanning mutagenesis (11 CDR sites). Each mutated CDR site involved sampling the wild-type residue and one to five of the most common natural diversity mutations. Degenerate codons were selected at each CDR site that maximized the natural diversity coverage and minimized the total number of mutations. It was not possible to sample the wild-type residue and the most common natural diversity mutations at each CDR site due to the limitations of degenerate codons. The resulting library (9.4 × 10^6^ variants) theoretically encodes all possible combinations of single and multiple CDR mutations (up to 14 mutations per V_H_H). The reported CDR site-specific natural diversity statistics are averaged values for human (V_H_) and camelid (V_H_H) variable domains, as reported in the abYsis database (55). Boxed amino acids correspond to the selected natural diversity mutations.

We next sought to design a single antibody library with mutations in N2 at permissive sites in CDR2 and CDR3. We aimed to accomplish multiple objectives in our library design. First, we limited the library size to ~10^7^ variants to enable 10-fold oversampling of the library using yeast surface display given that our typical yeast transformation efficiencies are ~10^8^ transformants. Second, we aimed to generate a single library with all possible combinations of wild-type residues as well as single and multiple mutations at the 11 permissive sites in CDR2 and CDR3 as well as at three additional sites not tested during alanine mutagenesis (A49, A94, and K95). This limits the number of possible mutations at each CDR site to typically one to two mutations in addition to the wild-type residue. Third, we sought to sample mutations that most closely correspond to those observed in the CDRs of natural antibodies in a site-specific manner. To accomplish this, we used the AbYsis database to identify the most common amino acids in camelid V_H_H and human V_H_ domains at each site in CDR2 and CDR3 (55). We used an average site-specific amino acid frequency for camelid and human domains at each CDR site given that there are many more sequences for human domains than for camelid domains. Fourth, we aimed to use inexpensive primer synthesis methods to generate the libraries encoded by standard degenerate codons. Therefore, we sought to identify degenerate codons at each CDR site that encoded the wild-type residue and ~1–5 additional residues that maximize the coverage (sum of individual site-specific amino acid frequencies) of the combined camelid and human natural diversity at each site (Figure 3).

**Figure 4:**
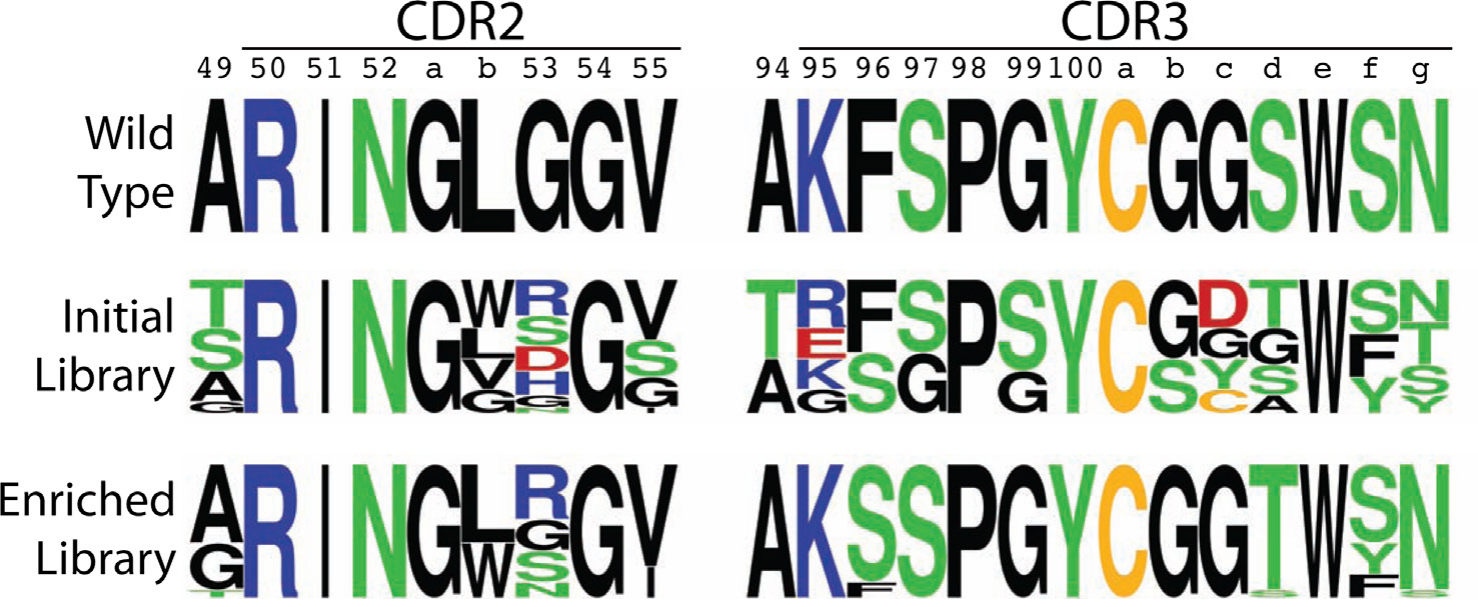
Amino acid logo summary of initial and enriched V_H_H libraries relative to the wild-type N2 V_H_H. The logo plots for the mutated portions of CDR2 and CDR3 were generated from sequencing results for 22 (initial library) and 17 (enriched library) V_H_H variants. The CDR sequences are defined using Kabat numbering, and the logos were generated using a web application (http://weblogo.berkeley.edu).

Based on these four key objectives, we designed the library shown in Figure 3 and generated it using the process outlined in Figure S3 in Supplementary Material. The library contains 9.4 × 10^6^ unique variants and includes wild-type residues at each position as well as all possible combinations of single and multiple mutations at 14 sites in CDR2 and CDR3. We sequenced several (22) members of the initial library, and the results are summarized in Figure 4 and Figure S4 in Supplementary Material. All variants were found to be unique and contained mutations according to the proposed library design.

### Sequence Analysis of V_H_H Libraries after Sorting for Enhanced Antigen Binding

The library of antibody variable domains was displayed on the surface of *S. cerevisiae* and screened for variants with increased affinity for the α-synuclein peptide. The sorting process involved five rounds of selection *via* magnetic-activated cell sorting (MACS) with progressively reduced concentrations of α-synuclein peptide (starting at 50 nM peptide and ending at 5 nM) and one additional round of selection *via* fluorescence-activated cell sorting (FACS) (20 nM peptide). The sorting process was continued until the antigen binding of the library was increased by at least fivefold relative to wild type, as judged by flow cytometry. Selections were performed in a buffer (PBS) that contained both BSA (1 mg/mL) and milk (1% w/v). We have found previously that antibody selections in complex environments (e.g., buffers supplemented with milk) lead to identification of antibodies with improved specificity (56).

The enriched V_H_H library was sequenced after sorts 5 and 6, and 17 unique variants were identified and further analyzed (based on sequencing 23 clones) with 1–6 mutations in CDR2 and CDR3. Sequence logos in Figure 4 summarize the general enrichment of amino acids in the CDRs, while the amino acid enrichment ratios are given in Figure S5 in Supplementary Material and the CDR sequences are given in Figure S6 in Supplementary Material. Most of the sites in CDR2 and CDR3 (11 out of 14) displayed either intermediate or strong preference for the wild-type residue (Figure 4). However, three sites (53 in CDR2, 96 and 100d in CDR3) either displayed similar preference for mutations as the wild-type residue (Arg, Gly, Ser, and Asn at position 53) or strong preference for a specific mutated residue (Ser at position 96 and Thr at position 100d). It is also notable that the four positions that were varied in CDR2 did not display strong preference for any single amino acid, while almost every residue in CDR3 (9 out of 10) displayed strong preference for a single residue. This result is unexpected based on alanine scanning mutagenesis, as the identified sites in CDR3 appeared to be as permissive (or even more permissive) to mutagenesis than those identified in CDR2.

### Identification of Affinity-Matured Variable Domains with High Stability and Specificity

**Figure 5:**
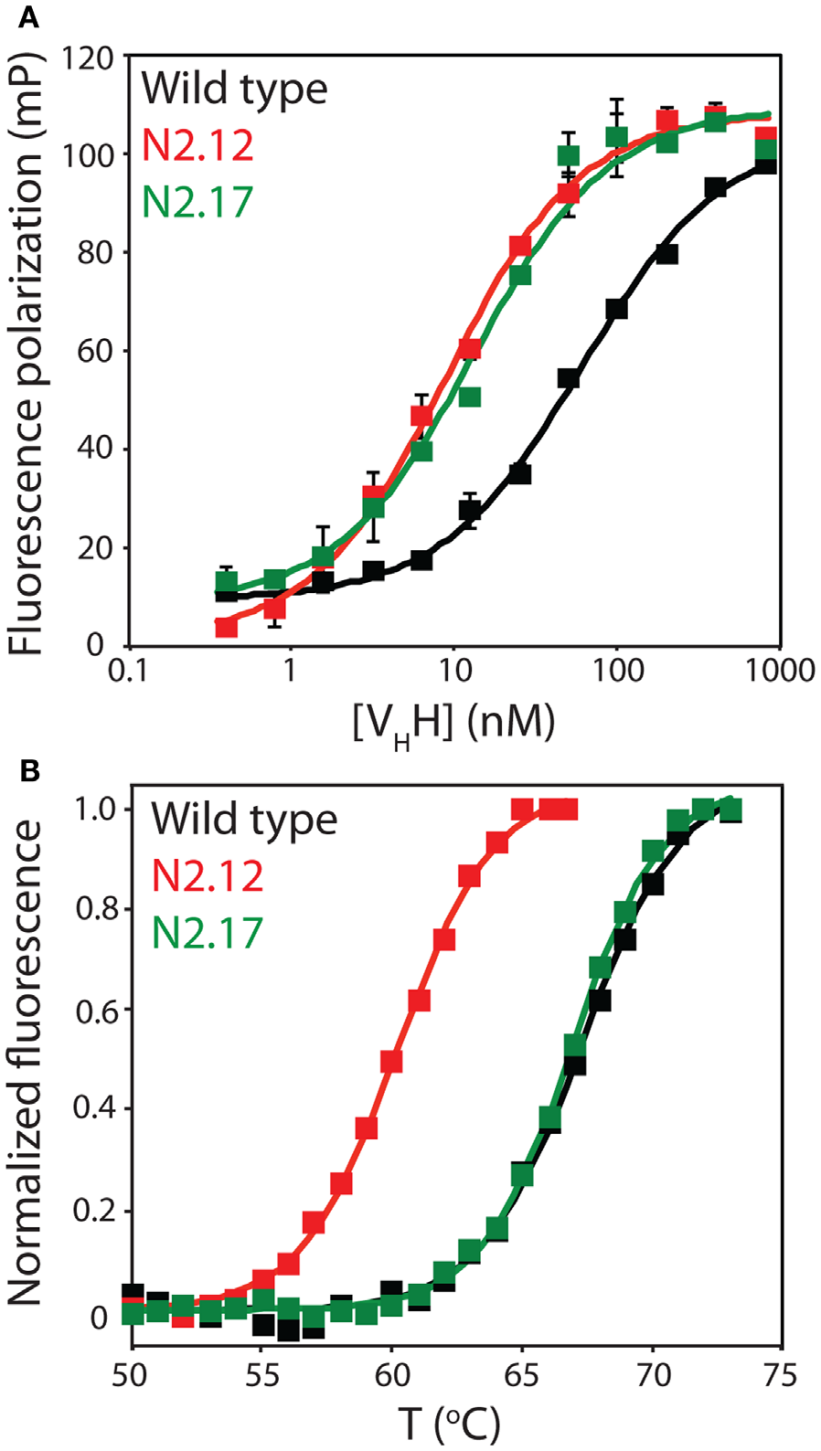
Evaluation of the affinity and stability of select V_H_H mutants that were enriched after library sorting for improved antigen binding. **(A)** Fluorescence polarization analysis of V_H_H binding to labeled antigen (2 nM TAMRA-labeled α-synuclein peptide). The analysis was performed in a PBS buffer supplemented with BSA (0.001%) and Tween 20 (0.001%). Three independent experiments were performed, and representative binding curves are shown for wild type (black), N2.12 (red), and N2.17 (green). Each point shown is the average of two repeats and the error bars are SD. Data were fit with a binding model that accounted for the fact that the V_H_H antibodies were not in excess of the antigen at some of the V_H_H concentrations (57). **(B)** Extrinsic fluorescence analysis of apparent V_H_H unfolding as a function of temperature. The fluorescence data were obtained using an extrinsic dye (Protein Thermal Shift dye, Life Technologies). Three independent experiments were performed, and representative melting curves are shown for wild type (black), N2.12 (red), and N2.17 (green). The data were background subtracted using background signals obtained without antibody. Next, the fluorescence data were subtracted by the relatively low signal at 50°C, and divided by the maximum fluorescence signal (after the maximum signal was subtracted by the signal at 50°C). Finally, the pre- and post-transition regions of the normalized fluorescence data were flattened using linear fits.

To evaluate the effectiveness of the affinity maturation process, we next expressed and purified the unique V_H_H variants that were identified in the enriched library. The variable domains expressed at levels (purification yields of 0.1–2.0 mg/L) that were generally similar to wild type (1.0 mg/L), and also displayed purities similar to wild type (Figure S7 in Supplementary Material). We first used fluorescence polarization to evaluate the affinities of the variable domains for the α-synuclein peptide (Figure 5A). The equilibrium dissociation constant for the wild-type N2 variable domain (*K_D_* of 57.6 ± 9.0 nM) was approximately threefold lower than the previously reported value (*K_D_* of 190 ± 30 nM) that was measured by isothermal calorimetry (52).

We chose to characterize two V_H_H domains in more detail (N2.12 and N2.17). Both variable domains displayed improved affinity (*K_D_* of 7.6 ± 0.4 nM for N2.12 and 13.2 ± 4.8 nM for N2.17 relative to 57.6 ± 9.0 nM for wild type; Figure 5A). Interestingly, the improved affinity of the N2.12 variant came at the cost of reduced stability (apparent *T_m_* of 59.7 ± 0.3°C relative to 67.8 ± 0.3°C for wild type; Figure 5B). By contrast, the N2.17 variant displayed similar stability as wild type (66.9 ± 0.1°C for N2.17 relative to 67.8 ± 0.3°C for wild type; Figure 5B). This finding demonstrates that our affinity maturation method can be used to identify antibody variable domains such as N2.17 with increased affinity without significant reduction in stability despite the common observation of affinity/stability trade-offs (such as those observed for N2.12) during affinity maturation (51, 58).

**Figure 6:**
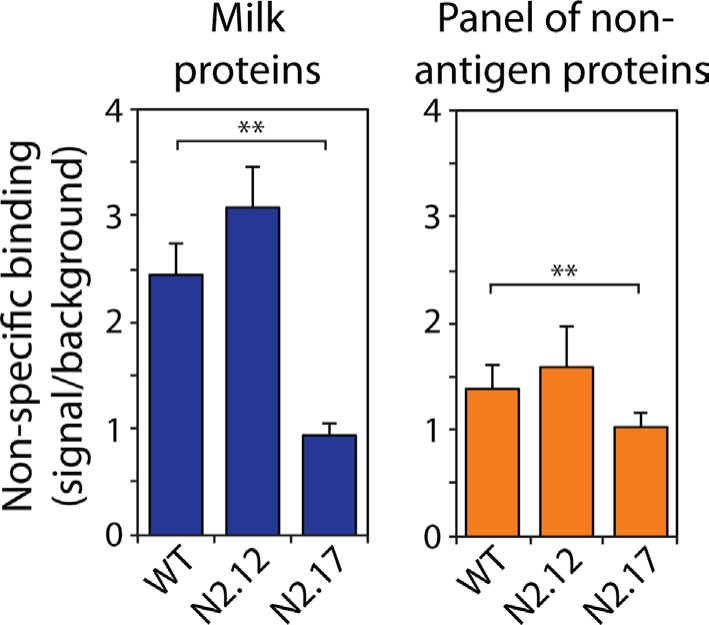
Analysis of non-specific binding for wild-type and affinity-matured V_H_H domains. Non-specific binding of V_H_H variants was evaluated using well plates coated with milk proteins (left) and a panel of six non-antigen proteins (right). The non-specific binding analysis was performed at an antibody concentration of 1,000 nM. The reported non-specific binding values are the signals for antibody binding to well plates coated with milk proteins or other non-antigen proteins divided by the background signal without primary antibody (V_H_H). The reported binding values (right) are the averages for six non-antigen proteins (ovalbumin, BSA, KLH, ribonuclease A, avidin, and lysozyme). The values are averages of three independent experiments, and the error bars are SD. A two-tailed Student’s *t*-test was used to determine statistical significance [*p*-values < 0.01 (**)].

We also evaluated the specificity of the N2.12 and N2.17 V_H_H domains to evaluate if gains in affinity were offset by reductions in specificity (Figure 6; Figure S8 in Supplementary Material). A simple test of non-specific interactions is to evaluate the propensities of antibodies to interact with well plates coated with different types of non-antigen proteins (milk proteins and a panel of six non-antigen proteins in Figure 6 and Figure S8 in Supplemental Material) at relatively high antibody concentrations (~1 μM). Interestingly, the N2.17 variant displays significantly lower non-specific interactions than wild type (*p*-values of 0.003 for milk proteins and 0.009 for six non-antigen proteins), while the N2.12 variant displays similar non-specific binding as wild type (*p*-values of 0.129 for milk proteins and 0.342 for non-antigen proteins). These results demonstrate that the affinity-matured V_H_H domains display similar or improved specificity relative to wild type.

**Figure 7:**
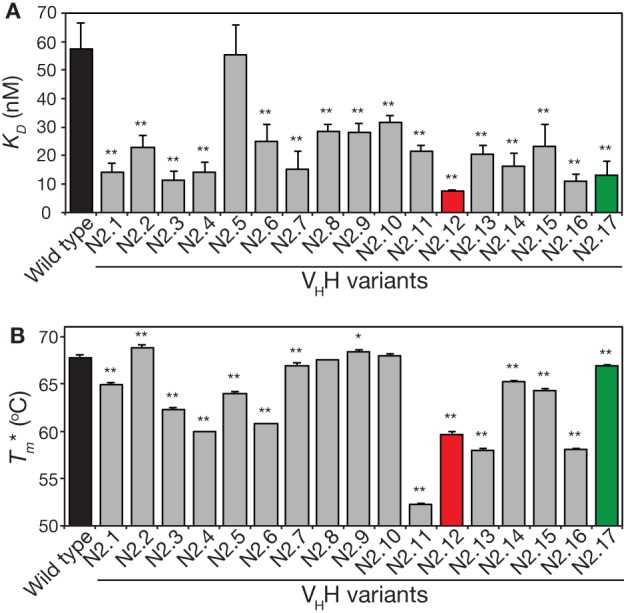
Analysis of affinity and stability for the panel of V_H_H mutants that were isolated after library sorting for improved antigen binding. **(A)** Equilibrium dissociation constant (*K_D_*) values were measured using fluorescence polarization. **(B)** Apparent melting temperatures $\left(T_{m^{*}}\right)$ were measured using extrinsic fluorescence measurements as a function of temperature. In **(A,B)**, the measurements were performed as described in Figure 5, the values are averages for three independent experiments, and the error bars are SD. A two-tailed Student’s *t*-test was used to determine statistical significance [*p*-values < 0.05 (*) or 0.01 (**)].

We next analyzed the affinity and stability of the other 15 unique V_H_H variants that were isolated during the sorting process (Figure 7; Figures S9 and S10 in Supplementary Material). All but one of the variable domains (N2.5) displayed a statistically significant increase in affinity relative to wild type (*p*-values <0.01; Figure 7A). This suggests that our library design and selection strategies enable robust identification of variable domains with improved affinity. Interestingly, variants with the greatest improvements in affinity (at least threefold) contained at least three mutations and up to six mutations. This highlights the inherent limitations of attempting to identify variable domains with large increases in affinity using single mutations.

**Figure 8:**
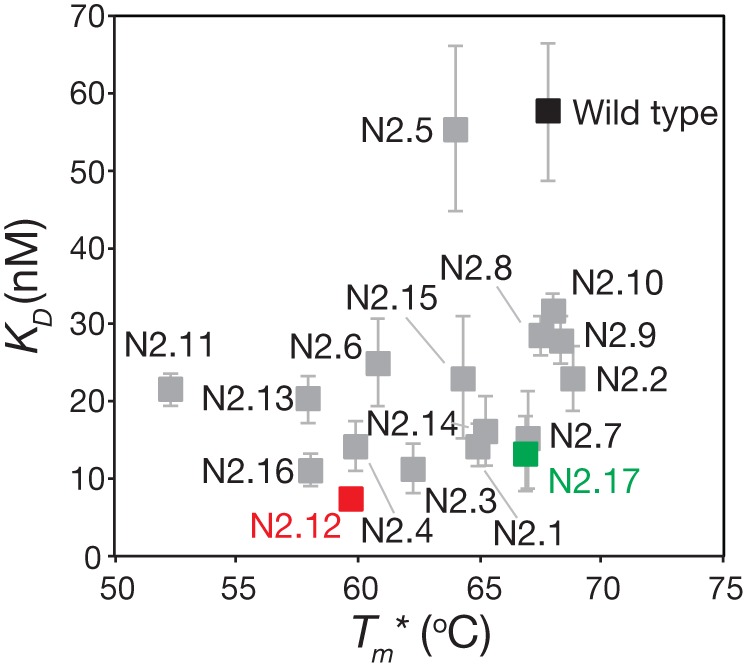
Comparison of the affinities and stabilities of the affinity-matured V_H_H variants. The equilibrium dissociation constants (*K_D_*) and apparent melting temperatures $\left(T_{m^{*}}\right)$ are from Figure 7. The values are averages of three independent experiments, and the error bars are SD (horizontal error bars for stability are smaller than the size of the data points).

The stability analysis of these variable domains also revealed interesting behaviors (Figure 7B). Most notably, the apparent stability of the V_H_H domains is much more variable than the affinity measurements. About one-third of variable domains (6 of 17) display similar stabilities as wild type (apparent melting temperature within 1°C of wild type). The variable domains with the largest reductions in apparent melting temperature (>7°C; N2.4, N2.11, N2.12, N2.13, and N2.16) had the highest number of mutations (5–6 mutations). A direct comparison of affinity versus stability for the V_H_H domains reveals a wide range of affinity/stability trade-offs (Figure 8).

### Origins of Affinity/Stability and Affinity/Specificity Trade-offs for Affinity-Matured V_H_H Domains

To better understand the origins of the strong and weak trade-offs between affinity and both stability and specificity for the selected V_H_H domains, we performed reversion mutational analysis for two of the variable domains (N2.12 and N2.17) to evaluate the impact of the acquired mutations on affinity, stability, and specificity. Six single reversion mutants were created for N2.12, while four single reversion mutations were created for N2.17. The purities of the reversion mutants were similar to wild type (Figure S11 in Supplementary Material).

**Figure 9:**
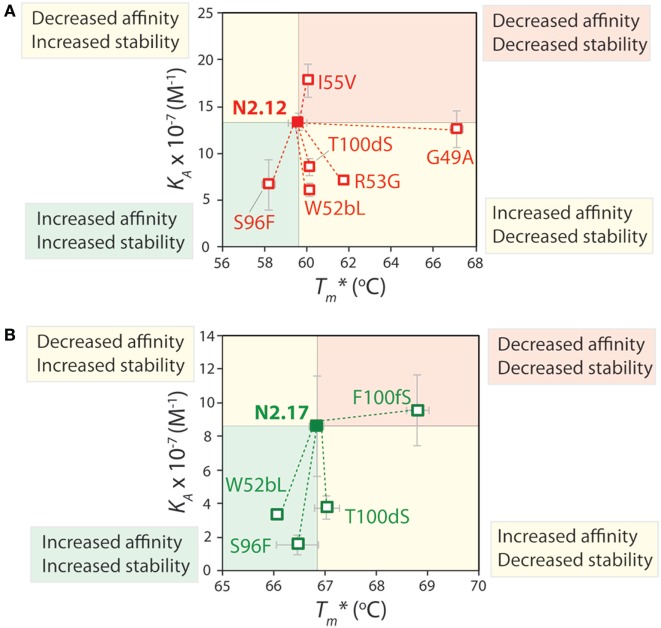
Mutational analysis of the contributions of specific V_H_H mutations to affinity and stability. Single reversion mutations were generated for two affinity-matured V_H_H variants [**(A)** N2.12 and **(B)** N2.17] to determine the contribution of each acquired mutation to affinity and stability. Values of the equilibrium association constant (*K_A_*) were measured using fluorescence polarization, and values of the apparent melting temperature $\left(T_{m^{*}}\right)$ were measured using extrinsic fluorescence measurements as a function of temperature. Reductions in either affinity or stability due to reversion mutations indicate that the original mutations acquired during affinity maturation contribute positively to either property. The values of *K_A_* and $T_{m^{*}}$ are averages from three independent experiments and the error bars are SD.

The affinity and stability measurements are summarized in Figure 9 and Figures S12 and S13 in Supplementary Material. In Figure 9, the affinity is reported as the equilibrium association constant (*K_A_*). Reversion mutations that reduced affinity and/or stability—which signifies that the original mutations increased affinity and/or stability—correspond to reduced *K_A_* or apparent melting temperature $\left(T_{m^{*}}\right)$ values. For the highest affinity variant identified in our studies (N2.12), one mutation (G49) is highly destabilizing and reversion to the wild-type residue (A49) results in a large increase in stability $\left(T_{m^{*}}\right)$ increases by 7.4°C; *p*-value of 2 × 10^−5^; Figure 9A) without a significant change in affinity (*p*-value of 0.67; Figure 9A). Surprisingly, this reversion mutant is the most desirable affinity-matured V_H_H domain that we obtained, as the large affinity enhancement (>7-fold) is achieved without compromising stability (*p*-value of 0.099 for comparison to wild type). This reversion mutational analysis also reveals that the affinity enhancement of N2.12 is largely due to four mutations (W52b, R53, S96, and T100d). The S96 mutation is particularly interesting because it contributes positively both to affinity and stability, as judged by the fact that the reversion mutation (F96) reduces both properties (*p*-values <0.03). By contrast, the R53 mutation increases affinity (*p*-value of 0.004) at the cost of stability (*p*-value of 0.001), and the W52b and T100d mutations increase affinity (*p*-values <0.005) without significantly impacting stability (*p*-values >0.1).

Reversion mutational analysis of the more stable V_H_H domain (N2.17) revealed key differences relative to the less stable N2.12 variant (Figure 9B). None of the four reversion mutations in N2.17 resulted in changes in apparent melting temperature >2°C. The most destabilizing N2.17 mutation was F100f, and the reversion mutation S100f increased stability to levels modestly higher than the wild-type N2 domain without a significant change in affinity relative to N2.17 (*p*-value of 0.74). The three key affinity mutations (W52b, S96, and T100d)—which were also observed in the less stable N2.12 domain—had little impact on stability (<1°C). These findings highlight that the affinity/stability trade-offs observed in our enriched library can be addressed either by screening a sufficient number of V_H_H variants or by performing reversion mutational analysis to identify destabilizing mutations that are not required for affinity.

**Figure 10:**
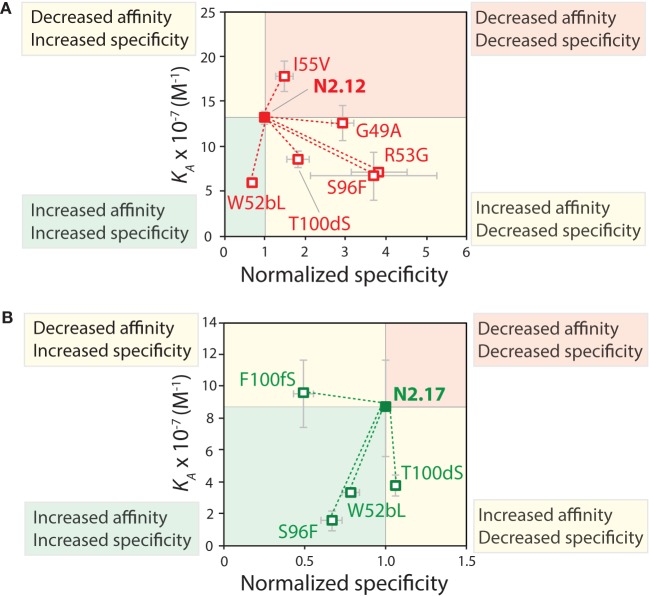
Mutational analysis of the contributions of specific V_H_H mutations to affinity and specificity. Single reversion mutations were generated for two affinity-matured V_H_H variants [**(A)** N2.12 and **(B)** N2.17] to determine the contribution of each acquired mutation to affinity and specificity. Values of the equilibrium association constant (*K_A_*) were measured as described in Figure 9. Normalized specificity was measured as the binding of the parent antibody to milk-blocked wells divided by that for the reversion mutant (parent/reversion mutant). Reductions in affinity or specificity for the reversion mutants indicates that the original mutations acquired during affinity maturation contribute positively to either property. The values of *K_A_* are averages from three independent experiments, and values for normalized specificity are averages of six replicates obtained from three independent experiments. The error bars are SD.

The specificity of the reversion mutants was analyzed by evaluating their relative propensity to interact with milk proteins (Figure 10). A decrease in the normalized specificity of a reversion mutant indicates that the original mutation has a positive impact on antibody specificity. The N2.12 variable domain—which possesses similar specificity as the wild-type N2 domain—acquired five mutations that decreased specificity (*p*-values <0.01; Figure 10A). However, N2.12 also acquired a single mutation (W52b) that increased specificity (*p*-value of 9.4 × 10^−6^; Figure 10A) and which appears to offset the negative effects of the other five mutations. Interestingly, the improved specificity of N2.17 relative to wild type appears to be due to three mutations that enhance specificity (W52b, S96, and F100f; *p*-values <1.1 × 10^−5^; Figure 10B). This analysis highlights that affinity-enhancing mutations can contribute both positively and negatively to antibody specificity, and that significant improvements in specificity can be due to the cumulative effects of multiple mutations.

### Computational Analysis of Natural Diversity Mutations That Enhance Affinity

**Figure 11:**
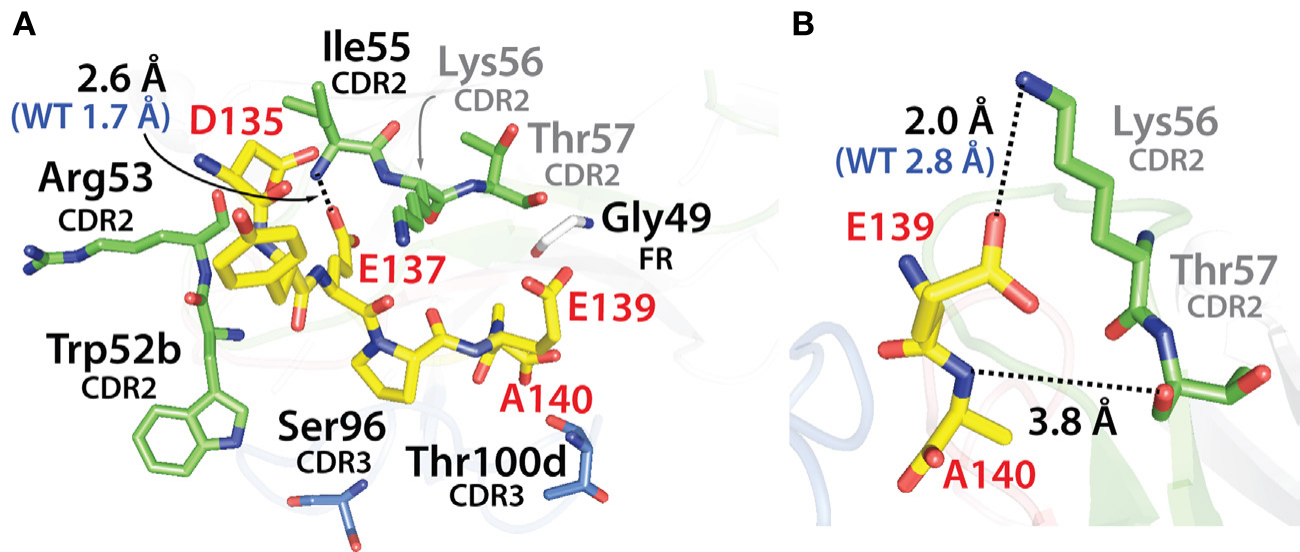
Analysis of the contributions of the acquired mutations in the N2.12 V_H_H antibody to enhanced affinity using computational models of the antibody–antigen complex. **(A)** Model of the N2.12 V_H_H in complex with the α-synuclein peptide. The six acquired CDR mutations are highlighted in black text, the wild-type residues are shown in gray, the nitrogen atoms are shown in blue, and the oxygen atoms are shown in red. Only one of the CDR mutations (Ile55) makes direct contact with the antigen, and the distance of this interaction is increased relative to wild type. **(B)** New or enhanced interactions between the N2.12 V_H_H and the α-synuclein peptide. Direct electrostatic interactions are shown with black dotted lines, and the distances are indicated in black for N2.12 relative to the original distances for wild type in blue (if there was a wild-type interaction). V_H_H residues are numbered according to Kabat.

To gain further understanding about how the selected mutations increased V_H_H affinity, we performed computational modeling of two of the mutant variable domains (N2.12 and N2.17). This was accomplished by introducing the corresponding mutations into the crystal structure of the wild-type N2 domain in complex with the α-synuclein peptide (PDB: 2X6M) and relaxing the structures *via* CHARMM force field energy minimization (59). The highest affinity domain we identified after library sorting (N2.12) contains six mutations that are located near but generally not in direct contact with the antigen (Figure 11A). The one exception is I55 in CDR2 (V55 in wild type), which forms a direct contact with E137 in the α-synuclein peptide *via* an interaction between the backbone amide in the antibody (I55) and carboxylate oxygen in the antigen (E137). However, this does not appear to explain the increased affinity of N2.12 because the mutation increases the interaction distance (2.6 Å) relative to wild type (1.7 Å). Instead, the increase in affinity for N2.12 appears to be due to indirect effects that involve enhancement of existing interactions as well as introduction of new interactions that involve wild-type CDR residues (Figure 11B). This includes an enhanced salt bridge between K56 (side chain nitrogen) in CDR2 and E139 (carboxylate oxygen) in the antigen. Moreover, a new electrostatic interaction is introduced between T57 (backbone carbonyl oxygen) in CDR2 and A140 (backbone amide nitrogen) in the antigen. The latter interaction appears to be mediated by a water bridge in both the crystal structure and energy minimized (relaxed) structure of the wild-type antibody-antigen complex (data not shown).

**Figure 12:**
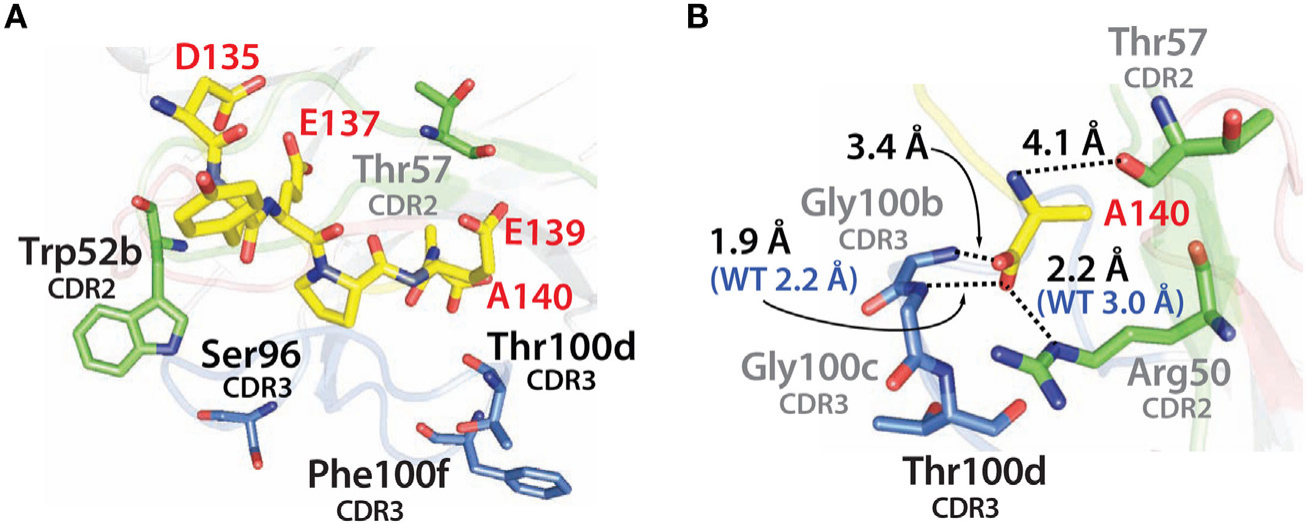
Analysis of the contributions of the acquired mutations in the N2.17 V_H_H antibody to enhanced affinity using computational models of the antibody–antigen complex. **(A)** Model of the N2.17 V_H_H in complex with the α-synuclein peptide. The four acquired CDR mutations are highlighted in black text. No CDR mutations make direct contact with the antigen. **(B)** New or enhanced interactions between the N2.17 V_H_H and the α-synuclein peptide. The labeling is the same as in Figure 11.

Similar findings were obtained by examining the modeled structure of the more stable N2.17 variant in complex with the α-synuclein peptide (Figure 12A). None of the four mutations make direct contact with the antigen. Instead, the gains in V_H_H affinity appear to be due to indirect effects involving wild-type CDR residues (Figure 12B), as observed for N2.12 (Figure 11B). We observe enhanced hydrophobic packing between G100b, G100c, and T100d in CDR3 with A140 in the antigen (Figure 12B). In addition, there are new direct electrostatic interactions between T57 (CDR2) and A140 (antigen) as well as G100b (CDR3) and A140 (antigen). Finally, two electrostatic interactions are enhanced, namely R50 (CDR2) with A140 (antigen) and G100c (CDR3) with A140 (antigen). This enhancement is due to A140 in the α-synuclein peptide moving deeper into the binding pocket of the V_H_H domain, which is mediated by structural rearrangement of the CDRs. These results are consistent with the general understanding that affinity maturation of antibodies involves subtle changes to the antigen-binding site and beneficial mutations often mediate their effects indirectly *via* structural changes that optimize interactions involving wild-type CDR residues (60–63).

## Discussion

This work identifies several key factors that impact the efficiency and robustness of antibody affinity maturation. First, we find that multiple mutations (>4) are necessary to achieve large (>5-fold) gains in affinity for the N2 V_H_H antibody. While there are obvious exceptions to our findings (1, 3, 5), they are generally consistent with previous findings that many single affinity-enhancing mutations cause relatively modest increases in affinity (24, 64–66). It is possible to identify and combine several single mutations that enhance affinity, but the collective effects of multiple mutations on antibody affinity are complex and often not additive (58, 62, 67, 68). Moreover, generating all possible combinations of single antibody mutations is a time-consuming process that involves multiple rounds of expression and affinity evaluation. It is also notable that the need for several mutations to achieve large increases in antibody affinity is likely at least part of the reason that it is particularly challenging to use computational methods for antibody affinity maturation (3, 24, 58, 67, 69). Accurate prediction of subtle structural changes caused by combinations of CDR mutations is notoriously difficult. Our natural diversity mutagenesis approach is attractive because it enables sampling of all possible combinations of single and multiple CDR mutations (~1–5 mutations per CDR site across 14 sites in this work) for rapid identification of antibody variants with large increases in affinity using a single antibody library.

There are multiple considerations related to our natural diversity mutagenesis approach that deserve further consideration. First, the primary problem during affinity maturation is obtaining mutations that increase affinity but reduce specificity. Our use of natural antibody diversity to guide library design—which has been reported previously in related ways by others (28–41, 70)—avoids overrepresentation of highly interactive residues that are likely to promote non-specific interactions. Many previous studies (including those from our own lab) have used NNN or NNK degenerate codons in antibody CDRs to identify affinity-enhancing mutations (58, 71–73). One of the limitations of this approach is that the frequency of sampling each amino acid is based on its corresponding codon frequency. In our experience, this is especially problematic for highly interactive residues such as arginine that have a large number of codons (up to six depending on the specific degenerate codon). By contrast, our library design infrequently sampled highly interactive residues, such as arginine (2 out of 14 CDR sites), tryptophan (1 out of 14 CDR sites), and phenylalanine (2 out of 14 CDR sites). In fact, one of the key affinity mutations in both N2.12 and N2.17 was F96S, which removed an aromatic residue and increased the hydrophilicity of CDR3.

It is also notable that our mutational approach was useful for identifying beneficial mutations in the highly variable CDR3 in addition to the less variable CDR2. Two of the key affinity mutations in both N2.12 and N2.17—F96S and S100dT—were in CDR3. The most common residues at many sites in CDR3 occur at relatively low frequency (13–21% for positions 95–100 g). Therefore, it was not obvious that sampling such a small number of natural diversity mutations (1–3 mutations per site for nine sites in CDR3) in such a highly diverse CDR would be sufficient to identify affinity-enhancing mutations. For example, the natural occurrence of the wild-type residue (Phe) at position 96 in CDR3 is 4% (combined human and camelid diversity), and we sampled only one mutation (Ser) at this site that is also relatively uncommon (9%) despite being more common than most other residues at this CDR3 site. Likewise, we sampled three mutations at position 100d in CDR3 (Gly, Ala, and Thr) that were all relatively uncommon (5–11%). Nevertheless, we identified a beneficial mutation (Thr) that occurs relatively infrequently (5%) at this site in CDR3. These results suggest that natural diversity mutations in CDR3—especially for affinity maturation—may be particularly useful for libraries aimed at isolating combinations of mutations that result in large increases in affinity without over enrichment in highly interactive residues that are likely to also mediate non-specific interactions.

Despite the strengths of our natural diversity mutagenesis approach, one obvious weakness is related to the use of inexpensive primer synthesis methods that rely on standard degenerate codons to generate libraries. This results in the limitation that some combinations of wild-type CDR residues and the most common natural diversity mutations are (i) not possible, (ii) require too many additional mutations to justify including them, and/or (iii) require inclusion of undesirable codons (e.g., those encoding cysteine or stop codons). While we allowed a cysteine mutation at one position (100c) to maximize natural diversity coverage, it is undesirable to include too many cysteine mutations due to complications associated with unpaired cysteines.

An example of the limitations of using degenerate codons to generate antibody libraries is related to position 52b in CDR2. The wild-type residue at position 52b is Leu, and the two most common residues at this position are Lys (29% based on combined camelid and human natural diversity) and Arg (22%). However, this requires sampling a minimum of six codons, which corresponds to a minimum of five residues and overrepresentation of arginine (two codons) to achieve natural diversity coverage of 56% (an average of ~9% per codon). Therefore, we sampled Gly, Val, and Trp in addition to the wild-type residue (Leu) at position 52b using four codons to achieve natural diversity coverage of 36% and similar average diversity per codon (9%). Likewise, the wild-type residue at position 96 in CDR3 is Phe. In order to sample Phe and the most common residue (Gly), this requires sampling a minimum of four codons that include Val (5%) and Cys (1.5%). Sampling these four residues would result in natural diversity coverage of 23% (an average of ~6% per codon). Instead, we sampled Ser in addition to Phe using two codons to achieve natural diversity coverage of 13% (an average of ~6% per codon). This approach allowed us to sample a similar amount of natural diversity per codon and eliminated the use of an undesirable codon (Cys). These examples highlight the limitations of using standard degenerate codons to achieve the highest possible coverage of natural diversity mutations. This limitation could be readily solved using more expensive trinucleotide synthesis methods.

Our results also demonstrate that affinity/stability trade-offs are common during antibody affinity maturation. We and others have previously found that CDR mutations that increase antibody affinity can be destabilizing (48, 49, 51). Indeed, several examples of natural antibodies have been reported that demonstrate how affinity-enhancing mutations can be destabilizing (48, 49). This destabilization is likely due to strain on the antibody framework that results from modifying the structure and chemistry of the antigen-binding site for increased affinity. Encouragingly, about one-third of our affinity-matured antibodies displayed little reduction in stability (<1°C) and we identified one of the highest affinity variants with similar stability as wild type after additional mutational analysis (N2.12 with A49; $T_{m^{*}}$ of 67.1 ± 0.3°C relative to 67.8 ± 0.3°C for wild type). Nevertheless, the fact that the highest affinity variants identified after library sorting were some of the most destabilized ones (e.g., N2.12 and N2.16) highlights the challenge of affinity/stability trade-offs during affinity maturation. One promising approach is to combine natural diversity mutations in the CDRs with those that naturally occur in the frameworks (74) to co-select for both affinity and stability mutations. We are currently in the process of evaluating this strategy to further improve the affinity maturation process for a wide range of single- and multidomain antibodies to isolate variants that possess high stability in addition to high affinity.

Another notable aspect of our findings relates to the impact of affinity-enhancing mutations on antibody specificity. Specificity is arguably the most difficult antibody property to maintain or enhance during affinity maturation (42–44). This is likely due to the natural tendency to accumulate highly interactive (solvent exposed) amino acids in antibody CDRs during affinity maturation that improve antigen binding but also promote non-specific interactions and reduced specificity. Indeed, we observed trade-offs between affinity and specificity for the N2.12 variant, as three of the four key affinity-enhancing mutations (R53, S96, and T100d) reduced specificity (Figure 10A). Interestingly, the N2.17 variant displayed reduced affinity/specificity trade-offs, as two (W52b and S96) of the three affinity-enhancing mutations also increased specificity (Figure 10B). The latter results are particularly notable because these same mutations (W52b and S96) also increased the stability of N2.17. It is also notable that the impacts of mutations on affinity and specificity were context dependent, as some mutations (e.g., S96) that increased affinity displayed opposite impacts on specificity (reduced specificity for N2.12 and increased specificity for N2.17). Despite these complexities, it will be important in the future to better define how CDR sequence and structure impacts antibody specificity because antibody specificity appears to be a key factor in differentiating approved antibody therapeutics from those in clinical trials (75).

## Conclusion

Our systematic approach for using natural antibody diversity to design libraries with combinations of single and multiple mutations with limited diversity at each CDR site is effective for increasing the affinity of a camelid V_H_H domain while maintaining or enhancing stability and specificity. These encouraging results will need to be evaluated for other types of single- and multidomain antibodies to evaluate their generality. It will also be important to develop computational methods to improve library design by optimizing natural diversity coverage while minimizing the number of mutations. This is relatively straightforward to perform at any given CDR site but it is more challenging to globally optimize with increasing numbers of CDR sites. Nevertheless, efforts in optimizing antibody library design are key to avoid oversampling abnormal CDR sequences that are unlikely to lead to high antibody stability and specificity in addition to high affinity. We expect that methods such as the ones we have demonstrated in this work will be useful for rapidly and systematically optimizing antibodies for a wide range of diagnostic and therapeutic applications.

## Experimental Methods

### Cloning and Library Construction

The wild-type N2 gene was created using PCR-based gene synthesis (76). The amino acid sequence of the N2 V_H_H domain (Figure 1) was obtained from the PDB (2X6M). A hexahistidine tag was added to the C-terminus of the V_H_H domain for purification. The gene was flanked with N-terminal *HindIII* and C-terminal *XhoI* restriction sites. The digested PCR product was then ligated into a bacterial expression vector (pET-17b, Novagen) that contained an N-terminal pelB sequence for periplasmic secretion. Single point mutations of N2 were generated *via* site-directed mutagenesis using *PfuUltra* II (600850, Agilent Technologies).

The N2 natural diversity library was created using overlap extension PCR to introduce mutations in portions of CDR2 and CDR3 (Figure S3 in Supplementary Material). Mutagenesis was performed using degenerate codons at 14 sites in CDR2 and CDR3 (Figure 3). The first step in library generation was to perform three PCRs. These included amplification of DNA fragments encoding the N-terminus of V_H_H domain to framework 2, CDR2 to framework 3, and CDR3 to the C-terminus of V_H_H domain. The DNA fragments overlapped each other by ~20 bases, which enabled the three DNA fragments to be combined in a final amplification step using terminal primers. The terminal primers contained flanking *NheI* and *SalI* restriction sites as well as 45 bases of homology on each end with the yeast display plasmid (pCTCON2).

The N2 natural diversity library genes were ligated into the yeast display plasmid and transformed into *S. cerevisiae* (EBY100) *via* homologous recombination. This process was performed as described previously (9) with minor modifications to increase transformation efficiency. These modifications include using more yeast cells (500 mL of EBY100 was grown to OD_600_ of 1.2) for a single library transformation, more DNA (nine preparations of 4 µg PCR product and 1 µg digested vector), and electroporation at higher voltage (2,500 V). After the yeast cells were allowed to recover, the yeast library was grown in SDCAA (500 mL of 20 g/L dextrose, 6.7 g/L yeast nitrogen base, 5 g/L casamino acids, 14.7 g/L sodium citrate, and 4.3 g/L citric acid) for 48 h, and aliquotted for storage at −80°C. The library transformation resulted in 2 × 10^8^ transformants. To assess the quality of the library, a small amount of the yeast library culture (1 mL) was miniprepped (Zymoprep II yeast miniprep kit, Zymo Research) and transformed into electroporation-competent bacterial cells (XL1-Blue, 200228, Agilent Technologies). Several (22) plasmids from the initial library were isolated and sequenced, and all were found to be unique.

### Yeast Surface Display and Library Screening

The yeast cultures were first grown at 30°C with agitation in SDCAA to an OD value of 1–2. To induce the expression of Aga2-V_H_H fusion proteins, the medium was switched to SGCAA (20 g/L galactose, 6.7 g/L yeast nitrogen base, 5 g/L casamino acids, 8.56 g/L NaH_2_PO_4_·H_2_O, and 6.76 g/L Na_2_HPO_4_·2H_2_O) and grown for 16 h at 30°C with agitation. The yeast medium was supplemented with ampicillin (100 µg/mL; BP1760-25, Thermo Fisher Scientific), kanamycin (100 µg/mL; BP906-5, Thermo Fisher Scientific), and penicillin-streptomycin (diluted to 1×; 15140122, Thermo Fisher Scientific).

The natural diversity library was sorted *via* five rounds of MACS and one round of FACS. For each sort, yeast were washed twice with PBS containing BSA (1 mg/mL; PBS-B) and resuspended in a solution containing the biotinylated α-synuclein peptide (biotin-GYQDYEPEA) and PBS-B supplemented with 1% milk (non-fat dry milk, PBS-BM). For the FACS sort, 1,000× diluted anti-c-myc chicken IgY antibody (A-21281, Life Technologies) was added to this mixture to detect V_H_H display. The yeast and α-synuclein peptide solution was mixed end-over-end at room temperature for 2–3 h. Next, the cells were washed once with PBS-B and sorted for antigen binding.

For MACS sorts, yeast cells were resuspended in PBS-B (5 mL) and mixed with Streptavidin MicroBeads (100 µL; 130-048-102, Miltenyi Biotec). After incubation on ice (10 min), the yeast cells were pelleted and resuspended in PBS-B and passed through a MACS separation column (130-042-401, Miltenyi Biotec). The column was connected to a MidiMACS separator magnet (130-042-302, Miltenyi Biotec) that was attached to a MACS MultiStand (130-042-303, Miltenyi Biotec). Next, the bound yeast cells were eluted by removing the column from the magnetic stand and flowing SDCAA (7 mL) through the column. The collected cells were then grown overnight in SDCAA (30°C) with agitation and subjected to additional rounds of sorting. For the FACS sort, yeast cells were resuspended in PBS-B (200 µL) with 100-fold diluted secondary reagents (Alexa Fluor 488-conjugated goat anti-chicken IgG, A-11039 and Alexa Fluor 647-conjugated streptavidin, S-32357; Life Technologies), and allowed to incubate on ice (5 min). The cells were washed once, analyzed, and sorted *via* flow cytometry (FACSAria, BD Biosciences). The enriched yeast cultures after sorts 5 and 6 were miniprepped and subcloned into a bacterial expression vector (pET-17b). Several (~10) plasmids from each sort were isolated and sequenced.

### Bacterial Expression and Purification

V_H_H domains were expressed in bacteria [BL21(DE3)pLysS, 200132, Agilent Technologies] using auto-induction media (200 mL) supplemented with ampicillin (100 µg/mL) and chloramphenicol (35 µg/mL) (77). After 48 h of growth at 30°C, the cultures were pelleted and the supernatants were incubated overnight (4°C, 80 rpm) with 3 mL of Ni-NTA beads (30230, Qiagen). The beads were then washed with PBS (150 mL), eluted at pH 3 (PBS), and neutralized to pH 7.4. The protein samples were centrifuged at 21,000 × *g* (5 min) and filtered (0.22 µm filter, SLGV013SL, Millipore). Next, the V_H_H domains were refolded *via* buffer exchange (Zeba spin desalting columns, 89893, Thermo Fisher Scientific) into 6 M GuHCl (pH 7.4). The antibody domains were allowed to equilibrate overnight (4°C) before being buffer exchanged into PBS (pH 7.4). Finally, the V_H_H domains were concentrated (3 kDa spin filters; UFC800324, EMD Millipore) and filtered again (0.22 µm filters). The concentrations of the V_H_H domains were measured *via* UV absorbance measurements at 280 nm. The extinction coefficients of the V_H_H domains were 27,180–32,680 M^−1^cm^−1^, which were calculated based on their amino acid sequences. The purity of the V_H_H domains was evaluated using SDS-PAGE analysis (WG1203BOX, Life Technologies), and the gels were stained using Coomassie dye (24615, Thermo Fisher Scientific).

### Antibody Affinity Analysis

The affinities of the N2 V_H_H and variants thereof were measured using fluorescence polarization. The V_H_H domains were prepared at a range of concentrations (0.8 nM–1.6 µM) and mixed (75 µL) with the α-synuclein peptide labeled with a tetramethylrhodamine (TAMRA) fluorophore (4 nM, 75 µL; Genemed Synthesis Inc.). The antibody–antigen mixtures were prepared in 96 well flat bottom black polystyrene plates (7605, ThermoFisher Scientific). The binding buffer was PBS supplemented with BSA [0.001% (w/v)] and Tween 20 [0.001% (v/v)]. Background wells were prepared that contained the same concentration of TAMRA-labeled α-synuclein peptide without antibody. The antibody–antigen mixtures were allowed to equilibrate at room temperature for 3 h. Fluorescence polarization was then measured (Infinite M1000 PRO, Tecan) at an excitation wavelength of 530 nm (5 nm bandwidth) and an emission wavelength of 582 nm (10 nm bandwidth).

The fluorescence polarization raw signals were background subtracted and two replicates were averaged for each antibody concentration. The average data were then fit to determine the *K_D_* value using a four-parameter model that accounts for the fact that the antibody is not in excess of antigen at some of the evaluated antibody concentrations:

$$\text{FP}={\text{FP}}_{\text{min}}+{\text{FP}}_{\text{max}}\frac{\left(\left[\text{Ab}\right]+\left[\text{Ag}\right]+K_{D}\right)-\sqrt{{\left(\left[\text{Ab}\right]+\left[\text{Ag}\right]+K_{D}\right)}^{2}-4\left[\text{Ab}\right]\left[\text{Ag}\right]}}{2\left[\text{Ag}\right]}$$

where FP is the measured fluorescence polarization value, FP_min_ is the minimum fluorescence polarization value, FP_max_ is the maximum fluorescence polarization value, [Ab] is the total V_H_H concentration, [Ag] is the total antigen concentration, and *K_D_* is the equilibrium dissociation constant. The equation was fit using the Microsoft Excel solver tool to minimize differences—namely the sum of squared differences—between the data and the model. At least three independent experiments were performed for each V_H_H antibody.

### Antibody Stability Analysis

The apparent stabilities of the V_H_H domains were determined using measurements of extrinsic fluorescence (Protein Thermal Shift dye, 4461146, Life Technologies) as a function of temperature. Protein Thermal Shift buffer (5 µL), V_H_H domains (12.5 µL of 0.08 µg/µL V_H_H), and Protein Thermal Shift dye (2.5 µL of 8× solution) were mixed in opaque 96-well PCR plates and sealed with foil (04729692001, Roche). The background samples were prepared with water (12.5 µL) instead of V_H_H domains. Thermal melts were performed using a LightCycler 480 real-time PCR instrument (Roche). The fluorescence (Ex: 558 nm, Em: 610 nm) was measured as the plate was heated from 37 to 95°C. Many (>60) acquisitions were collected per 1°C, and the heating rate was ~0.6°C/min.

The apparent melting temperatures of the V_H_H domains were determined by analyzing the first derivative of the fluorescence with respect to temperature. This involved fitting a second-order polynomial to the major peak and solving for the temperature at which the maximum occurred (or the minimum if the negative derivative is used). The reported melt curves were background subtracted using background signals obtained without antibody. Next, the fluorescence data were subtracted by the relatively low signal at 50°C and divided by the maximum fluorescence signal (after the maximum signal was subtracted by the signal at 50°C). Finally, the pre- and post-transition regions of the normalized fluorescence data were flattened using linear fits (58).

### Antibody Specificity Analysis

The specificities of the V_H_H domains were evaluated using two methods. The first method evaluated the propensity of the purified antibodies to bind to well plates coated with milk proteins. Transparent 384 well plates (MaxiSorp, 464718, ThermoFisher Scientific) were coated with milk [100 µL of 10% (w/v) milk in PBS with 0.1% (v/v) Tween 20; PBST] for 8 h and then washed with PBS. The V_H_H domains were diluted to 1,000 nM in PBST, added to the well plates and allowed to incubate overnight at room temperature. The well plates were then washed with PBS and secondary reagents were added to detect bound antibodies. The second method evaluated the propensity of the purified antibodies to bind to six immobilized non-antigens [ovalbumin (A5503, Sigma), BSA (BP9706, Fisher Bioreagents), KLH (H8283, Sigma), ribonuclease A (R6513, Sigma), avidin (A9275, Sigma), and lysozyme (L6876, Sigma)]. Non-antigen proteins were diluted in PBS (75 µL, 0.2 mg/mL) and immobilized in separate wells at 37°C for 1 h in 384 well plates. The wells were subsequently washed with PBST. Variable domains (1,000 nM, 25 µL) in PBS with 1 g/L BSA and 0.1% (v/v) Tween 20 were added to the well plates and allowed to incubate at room temperature for 2 h.

Detection of bound V_H_H was performed similarly for both specificity tests. Secondary antibody (25 µL of 1,000× diluted anti-6X His tag antibody; ab18184, Abcam) in PBST was added, allowed to incubate for 1 h, and then washed with PBS. Next, the well plates were incubated with diluted horseradish peroxidase-conjugated goat anti-mouse IgG (25 µL of 1,000× dilution; 32430, Thermo Fisher Scientific) in PBST for 1 h and then were washed with PBS. The bound antibody was detected by adding substrate (25 µL of 1-Step Ultra TMB-ELISA, 34028, Thermo Fisher Scientific), quenching after 20–40 min (25 µL of 2 M H_2_SO_4_) and measuring the absorbance values at 450 nm (Tecan Safire^2^ plate reader). Normalized binding signals were calculated as signal divided by background, and the background values were absorbance measurements without primary (V_H_H) antibody.

### Computational Modeling

The V_H_H-antigen crystal structure (PDB: 2X6M) was energy minimized using the CHARMM force field and the adopted basis Newton–Raphson routine (78). We applied the Newton–Raphson algorithm to a subspace of the coordinate vectors that were sampled by the displacement coordinates (during each iteration) with the objective of minimizing the energy of the complex. This enabled the rate of change of the gradient vectors to be computed and coupled with a subsequent eigenvector analysis to avoid saddle points (metastable energy states). At every Newton–Raphson iteration, the residual gradient vector was calculated and a steepest descent step was added to the Newton–Raphson step. This was done to incorporate a new direction into the basis set to avoid metastable states and find the shortest trajectory toward the atomic coordinates corresponding to the minimum potential energy of the complex.

Computational alanine scanning mutagenesis was performed in a similar manner as described previously (79). Python scripts were written for a new OptMAVEn module to compute the difference between binding energies of the N2 single alanine mutants (which were energy-minimized) and wild-type N2 (2X6M). Binding energy calculations were performed using the conformation-dependent binding energy function as used in the Robetta full-chain protein structure prediction server (80, 81).

Structural models of two affinity-matured V_H_H variants (N2.12 and N2.17) in complex with antigen were also generated. These structures were simulated alongside the energy-minimized wild-type complex. We created the N2.12 and N2.17 variants using the Mutator program of IPRO suite of programs (82). This approach uses the residue positions and mutations as input, and it performs backbone perturbation, rotamer repacking and energy minimization. A mixed-integer linear programming optimization step was performed to systematically identify the optimal rotamer combination of the new residues at the mutation sites and residues within 4.5 Å (83). This was done to prevent energetically unfavorable steric clashes upon mutation. We performed ensemble structure refinements to establish favorable Lennard-Jones interactions in addition to eliminate severe steric repulsions. The N2.12 and N2.17 variants were visualized in complex with antigen using PyMOL (version 1.8, Schrödinger). Shell scripts were written to identify direct and indirect polar contacts between the antigen (α-synuclein residues DYEPEA) and V_H_H variants. Only contacts within 5 Å were analyzed.

## Author Contributions

KT, PT, RC, TL, and CM designed the research; KT and SS performed experiments; RC and TL performed computational analysis; SL performed bioinformatics analysis; and KT, RC, CM, and PT wrote the paper.

## Conflict of Interest Statement

PT has received consulting fees and/or honorariums for presentations of this and/or related research findings at MedImmune, Eli Lilly, Bristol-Myers Squibb, Janssen, Merck, Genentech, Amgen, Pfizer, Adimab, Abbvie, Abbott, DuPont, Schrödinger, and Novo Nordisk. All other authors declare that the research was conducted in the absence of any commercial or financial relationships that could be construed as a potential conflict of interest.
