# Supplementary material for: Facile Affinity Maturation of Antibody Variable Domains Using Natural Diversity Mutagenesis
Source: Front Immunol. 2017 Sep 4;8:986. doi: 10.3389/fimmu.2017.00986 (PMC5591402; doi:10.3389/fimmu.2017.00986)
Supplement: Supplementary file 1 [file Presentation_1.PDF]

Supplemental Data

Facile affinity maturation of antibody variable domains using natural diversity mutagenesis

Kathryn E. Tiller, Ratul Chowdhury, Tong Li, Seth Ludwig, Sabyasachi Sen, Costas Maranas, Peter M. Tessier

Table S1. Computational alanine scanning mutagenesis of the CDRs of the N2 variable domain. The change in interaction energy between N2 and the α-synuclein peptide due to alanine mutation ($\Delta\Delta G_{obs}$) was calculated for residues in CDR2 and CDR3. Residues identified as important for binding (and not permissive to mutagenesis) are highlighted in red ($\Delta\Delta G_{obs}$ > 1.0 kcal/mol). Some alanine mutants in each CDR failed to change the interaction energy and are not reported.

CDR2

Residue	$\Delta\Delta G_{obs}$ (kcal/mol)
R50	0.23
I51	-0.02
N52	3.3
L52b	0.03
V55	0.01
K56	1.7
T57	-0.01
A58	0.01
Y59	0.01

CDR3

Residue	$\Delta\Delta G_{obs}$ (kcal/mol)
K95	-0.02
F96	0.08
S97	-0.1
Y100	1.1
C100a	-0.13
S100d	0.1
W100e	1.1
S100f	0.01

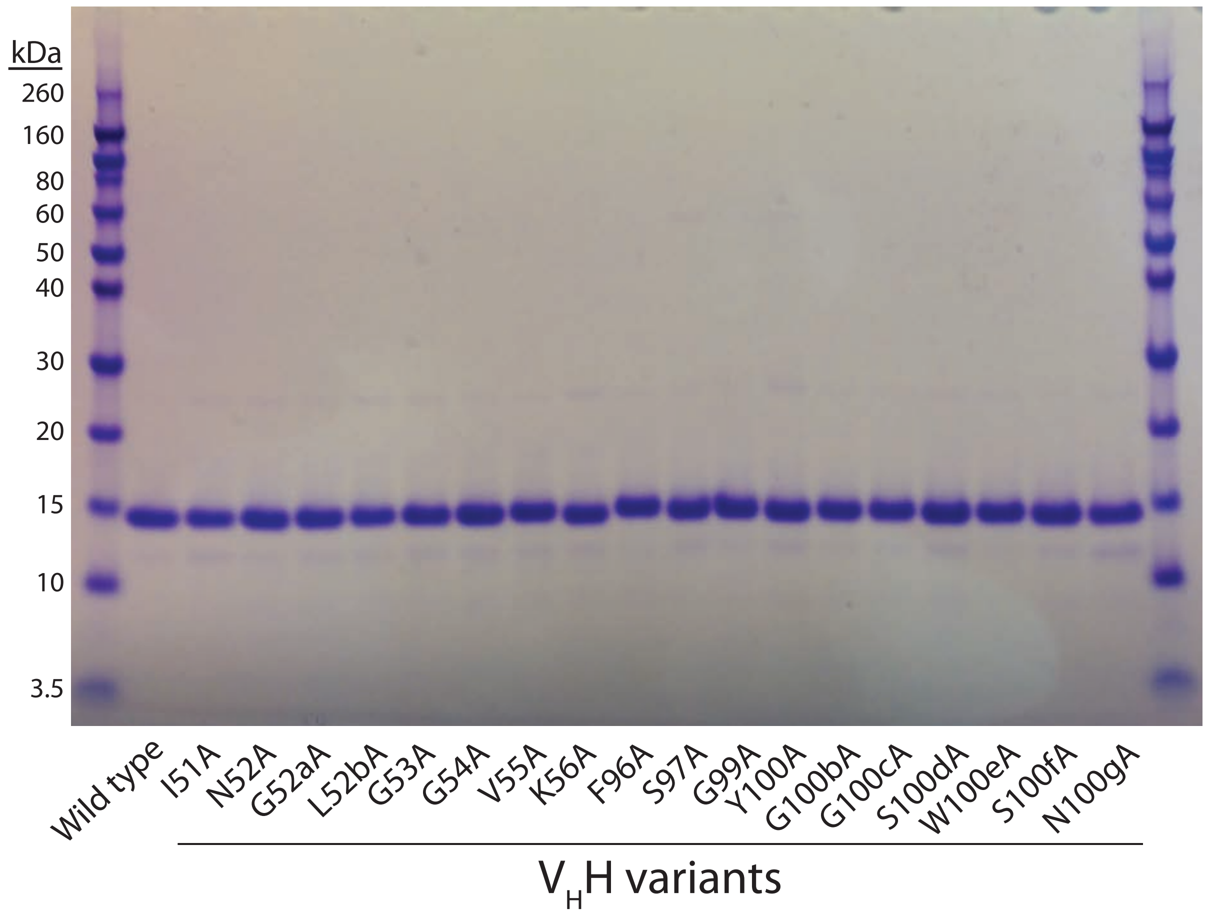

Figure S1. SDS-PAGE analysis of wild type and alanine mutants of the N2 V_HH antibody. V_HH domains (0.75 µg) produced in *E. coli* were analyzed using SDS-PAGE gels (10% Bis-Tris) that were stained with Comassie dye. The single alanine mutations are defined using Kabat numbering.

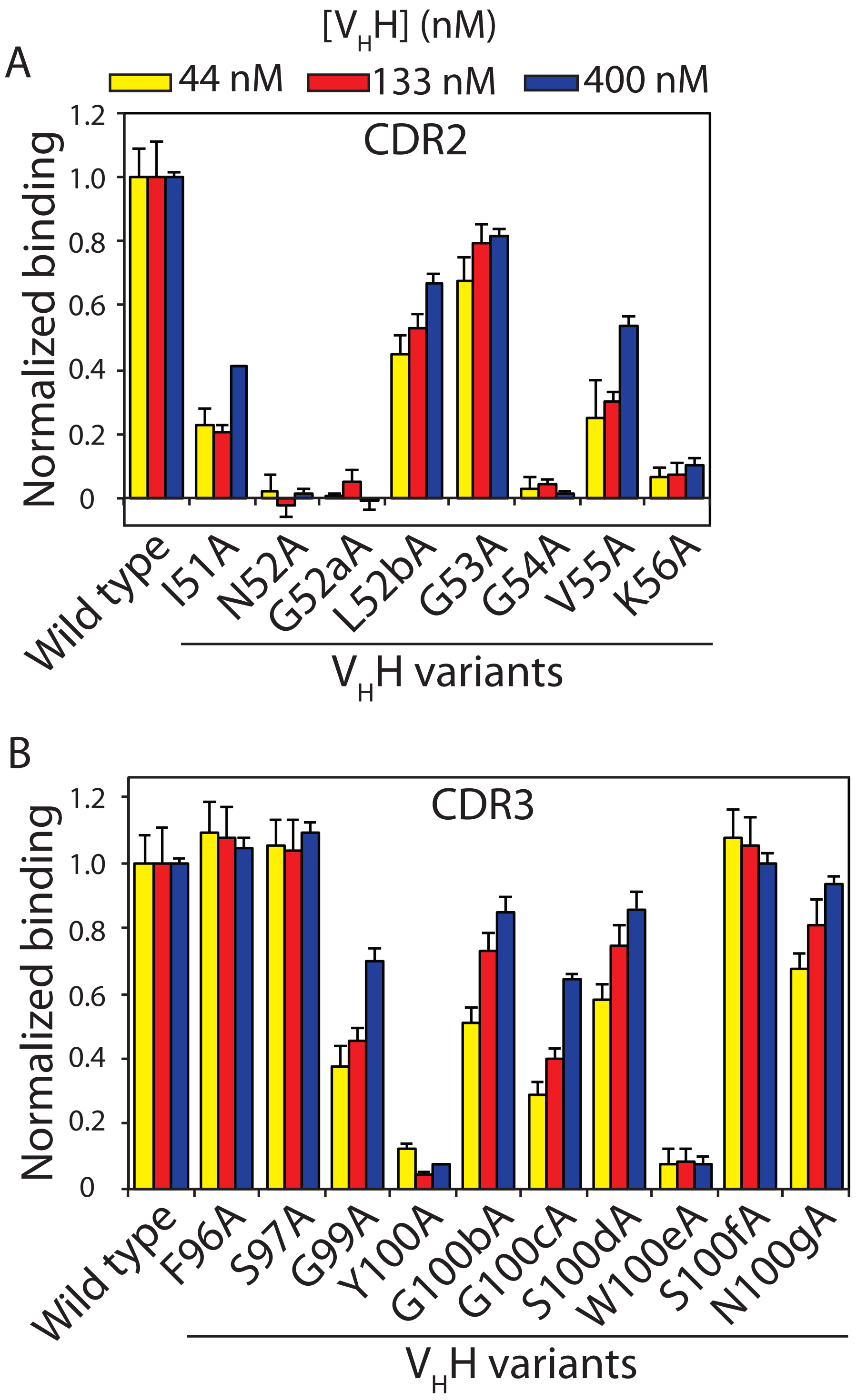

Figure S2. Identification of V_HH CDR residues involved in antigen binding via alanine scanning mutagenesis. The relative antigen binding of the V_HH variants (44, 133 and 400 nM) with single alanine substitution mutations in (A) CDR2 and (B) CDR3 was evaluated using fluorescence polarization (2 nM TAMRA labeled α-synuclein peptide). The measurements were obtained and processed as described in Figure 2. Error bars represent the standard deviations for three independent experiments.

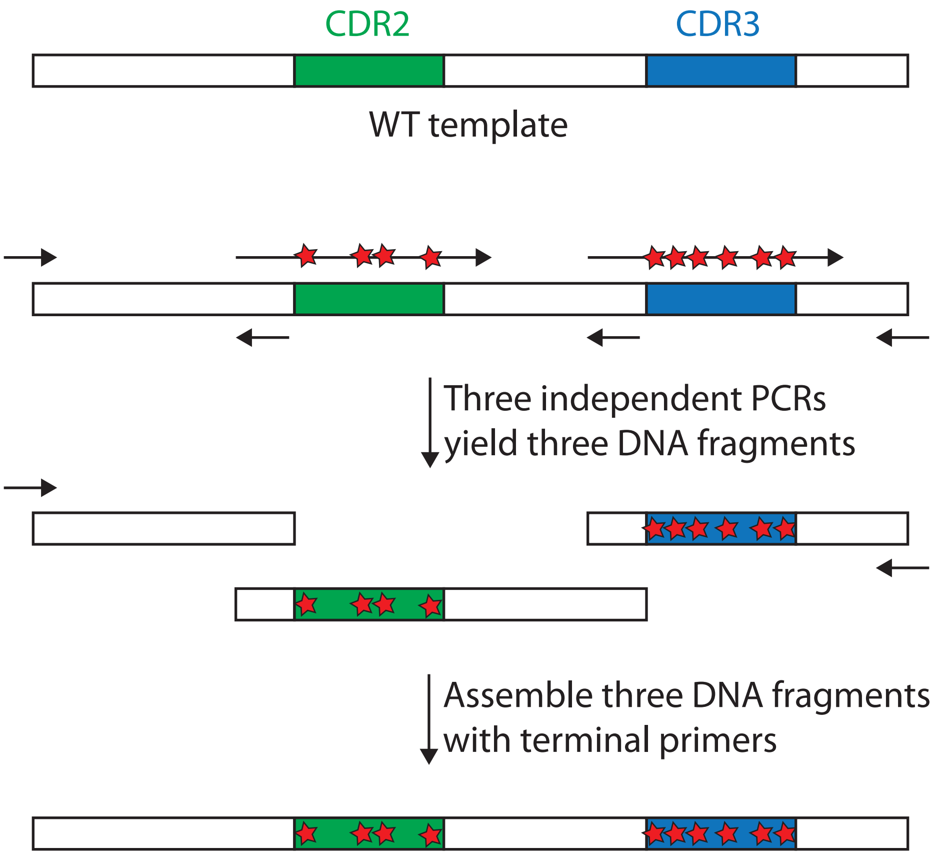

Figure S3. PCR process for creating the natural diversity library. The natural diversity library was created in two steps. The first step involved three PCR reactions with primers that encoded diversity at four positions in CDR2 and ten positions in CDR3 using degenerate codons (red stars). The second step assembled the three DNA fragments from the first PCR step to make a complete V_HH gene with targeted mutagenesis in CDR2 and CDR3.

CDR2

Position	49	50	51	52	52a	52b	53	54	55
Wild type	**A**	R	I	N	G	**L**	**G**	G	**V**
	**S** 25, 32%					**G** 25, 18%	**S** 17, 23%		**G** 25, 23%
	**A** 25, 23%					**V** 25, 23%	**D** 17, 18%		**S** 25, 27%
	**G** 25, 9%					**L** 25, 23%	**G** 17, 9%		**I** 25, 5%
	**T** 25, 36%					**W** 25, 36%	**N** 17, 5%		**V** 25, 46%
							**H** 17, 18%		
							**R** 17, 27%		

CDR3

Position	94	95	96	97	98	99	100	100a	100b	100c	100d	100e	100f	100g
Wild type	**A**	**K**	**F**	**S**	P	**G**	Y	C	**G**	**G**	**S**	W	**S**	**N**
	**A** 50, 41%	**G** 25, 18%	**S** 50, 46%	**G** 50, 46%		**G** 50, 32%			**G** 50, 59%	**Y** 25, 23%	**G** 25, 27%		**Y** 33, 27%	**Y** 25, 14%
	**T** 50, 59%	**E** 25, 23%	**F** 50, 55%	**S** 50, 55%		**S** 50, 68%			**S** 50, 41%	**G** 25, 23%	**S** 25, 23%		**F** 33, 32%	**S** 25, 23%
		**R** 25, 36%								**C** 25, 18%	**A** 25, 14%		**S** 33, 41%	**T** 25, 32%
		**K** 25, 23%								**D** 25, 36%	**T** 25, 36%			**N** 25, 32%

Figure S4. Summary of the predicted and observed amino acid frequencies at each mutated CDR site in the initial N2 V_HH library. The reported values in each box correspond to the expected (first number) and observed (second number) frequencies of each amino acid in the V_HH library. The observed amino acid frequencies are based on the sequences of 22 V_HH variants in the initial library.

CDR2

Position	49	50	51	52	52a	52b	53	54	55
Wild type	**A**	R	I	N	G	**L**	**G**	G	**V**
	**S** 0					**G** 0	**S** 1.0		**G** 0
	**A** 2.6					**V** 0	**D** 0		**S** 0
	**G** 3.9					**L** 2.6	**G** 2.6		**I** 5.2
	**T** 0.2					**W** 1.1	**N** 2.6		**V** 1.7
							**H** 0		
							**R** 1.5		

CDR3

Position	94	95	96	97	98	99	100	100a	100b	100c	100d	100e	100f	100g
Wild type	**A**	**K**	**F**	**S**	P	**G**	Y	C	**G**	**G**	**S**	W	**S**	**N**
	**A** 2.4	**G** 0	**S** 1.9	**G** 0		**G** 3.1			**G** 1.7	**Y** 0	**G** 0		**Y** 0.9	**Y** 0
	**T** 0	**E** 0	**F** 0.2	**S** 1.8		**S** 0			**S** 0	**G** 4.4	**S** 0.3		**F** 0.6	**S** 0.3
		**R** 0								**C** 0	**A** 0		**S** 1.4	**T** 0
		**K** 4.4								**D** 0	**T** 2.6			**N** 3.0

Figure S5. Summary of the amino acid enrichment ratios at each mutated CDR site in the V_HH library after sorting for enhanced antigen binding. Enrichment ratios were calculated by dividing the frequency of a given amino acid at a mutated CDR site relative to its corresponding frequency in the initial library.

```
                  CDR2             CDR3
        49    ab        94    100abcdefg
   WT:....ARINGLGGV...AKFSPGYCGGSWSN...
 N2.1:....ARINGWRGI...AKFSPGYCGGTWSN...
 N2.2:....ARINGLGGV...AKSSPGYCGGTWSN...
 N2.3:....GRINGWGGV...AKSSPGYCGGTWSN...
 N2.4:....GRINGLRGI...AKSSPGYCGGTWSN...
 N2.5:....ARINGLSGV...AKSSPGYCGGTWFS...
 N2.6:....GRINGLNGV...AKSSPGYCGGTWSN...
 N2.7:....ARINGLRGV...AKSSPGYCGGTWSN...
 N2.8:....ARINGLSGV...AKSSPGYCGGTWSN...
 N2.9:....ARINGLNGV...AKSSPGYCGGTWSN...
N2.10:...ARINGWGGV...AKFSPGYCGGSWSN...
N2.11:...TRINGWSGV...AKSSPGYCGGTWYN...
N2.12:...GRINGWRGI...AKSSPGYCGGTWSN...
N2.13:...GRINGLRGV...AKSSPGYCGGTWYN...
N2.14:...ARINGLRGV...AKSSPGYCGGTWYN...
N2.15:...ARINGLRGI...AKSSPGYCGGTWFN...
N2.16:...GRINGWSGV...AKSSPGYCGGTWYN...
N2.17:...ARINGWGGV...AKSSPGYCGGTWFN...
```

Figure S6. Sequences of the selected V_HH domains after sorting for enhanced antigen binding. The amino acid sequences are shown for the portions of CDR2 and CDR3 that were mutated, and the mutations are highlighted in red. The isolated V_HH domains contained one to six CDR mutations.

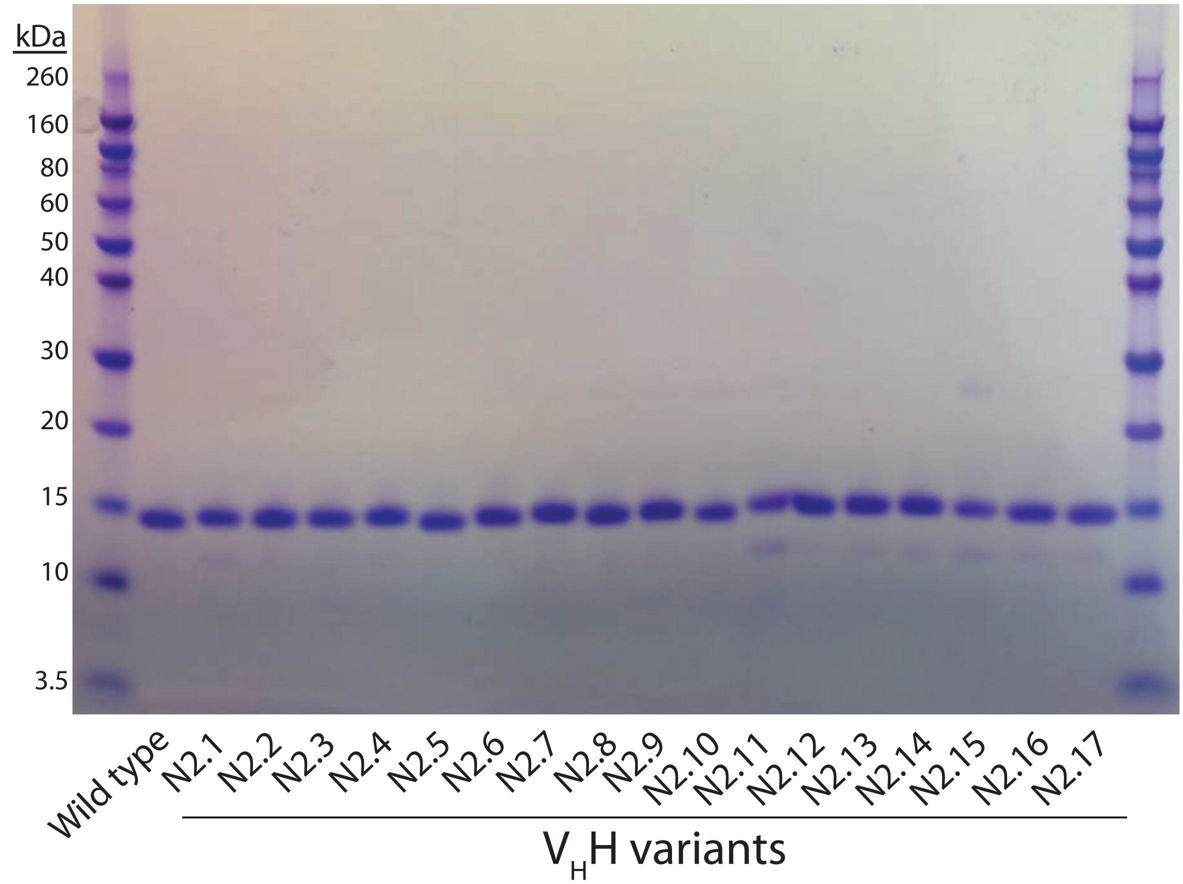

Figure S7. SDS-PAGE analysis of wild type and affinity-matured variants of the N2 V_HH antibody. The analysis was performed as described in Figure S1.

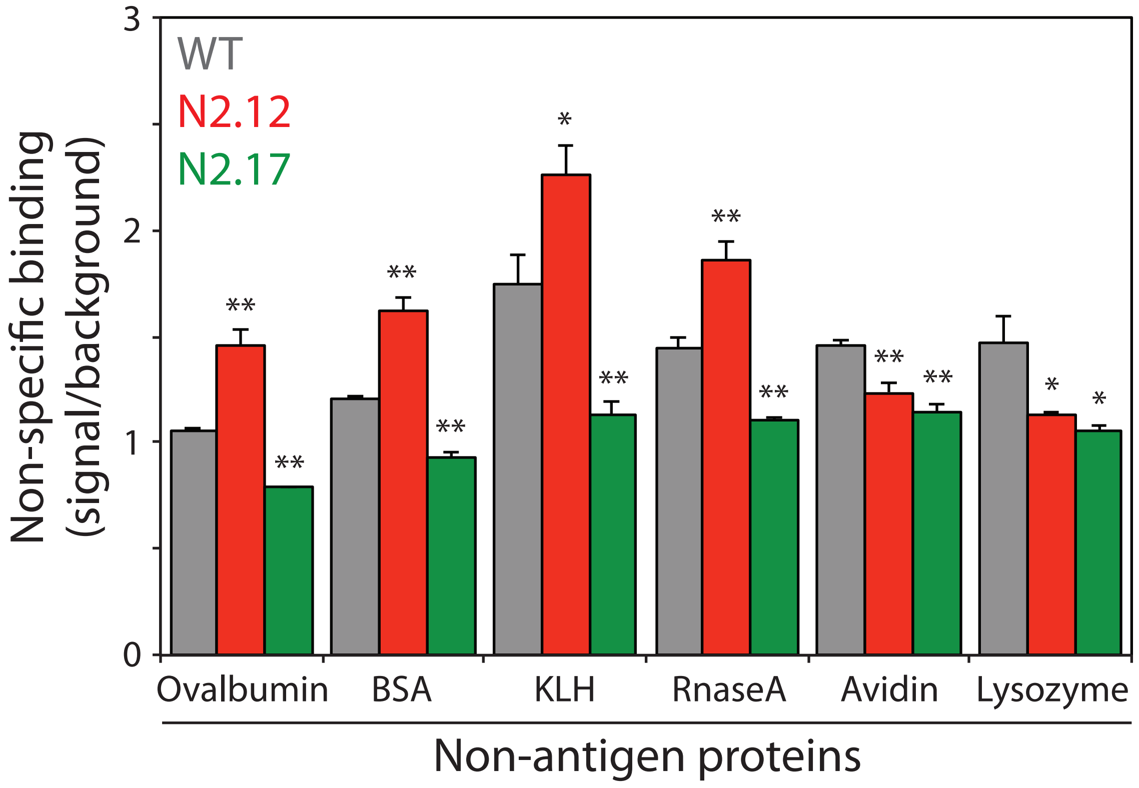

Figure S8. Analysis of non-specific binding for wild type and affinity-matured V_HH domains. Non-specific binding of V_HH variants was evaluated using well plates coated with a panel of six non-antigen proteins. The non-specific binding analysis was performed at an antibody concentration of 1000 nM. The reported non-specific binding values are the signals for antibody (V_HH) binding to well plates coated with a particular non-antigen protein divided by the background signal without primary antibody (V_HH). The values are averages of three independent experiments, and the error bars are standard deviations. A two-tailed Student's *t*-test was used to determine statistical significance [*p*-values < 0.05 (*) or 0.01 (**)].

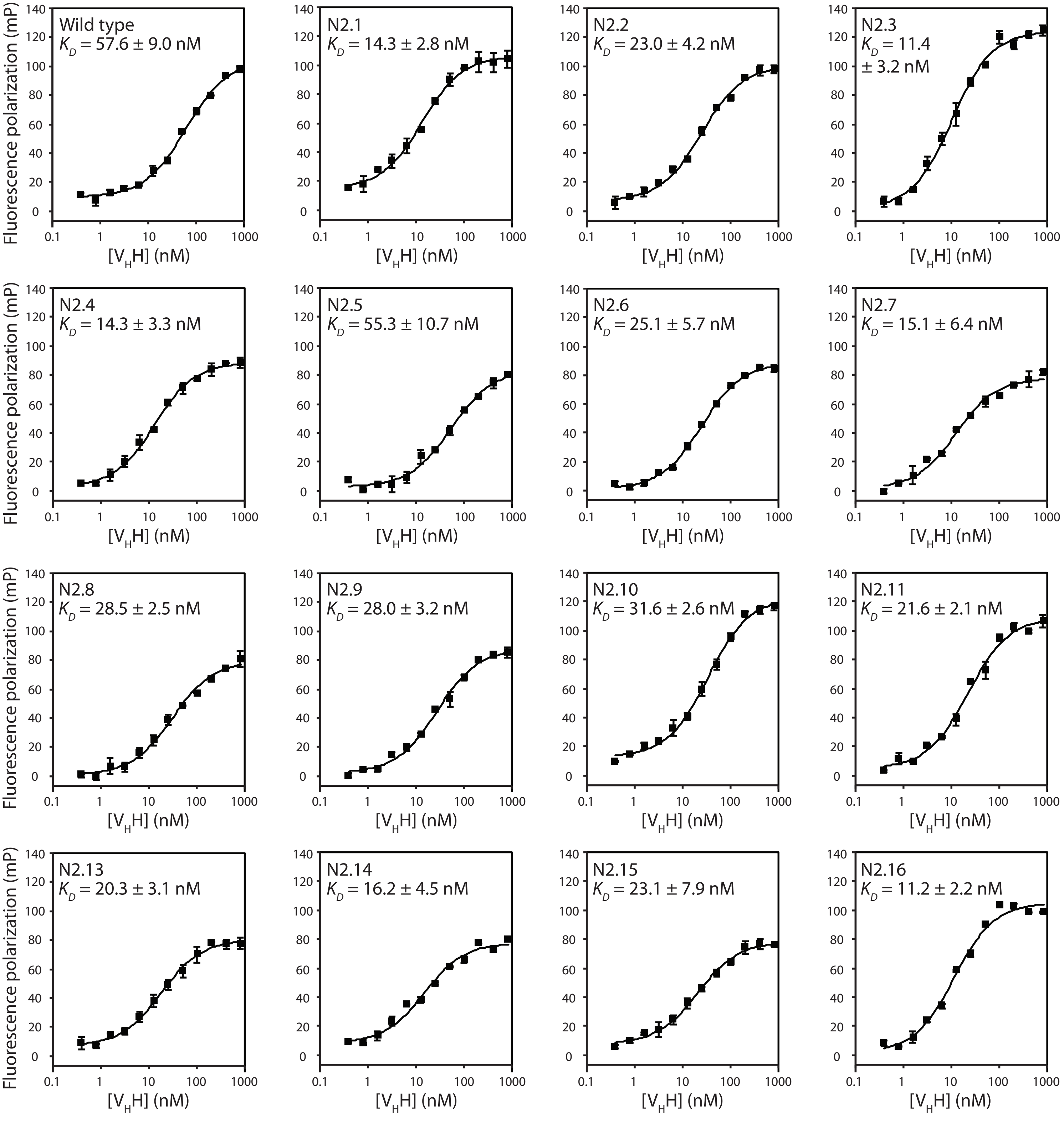

Figure S9. Summary of affinity measurements for the wild type and affinity-matured V_HH variants. Representative binding curves are shown for fluorescence polarization measurements of V_HH affinity. The affinity analysis was performed as described in Figure 5. The equilibrium dissociation constant (K_D) values are averages for three independent experiments, and the errors are standard deviations.

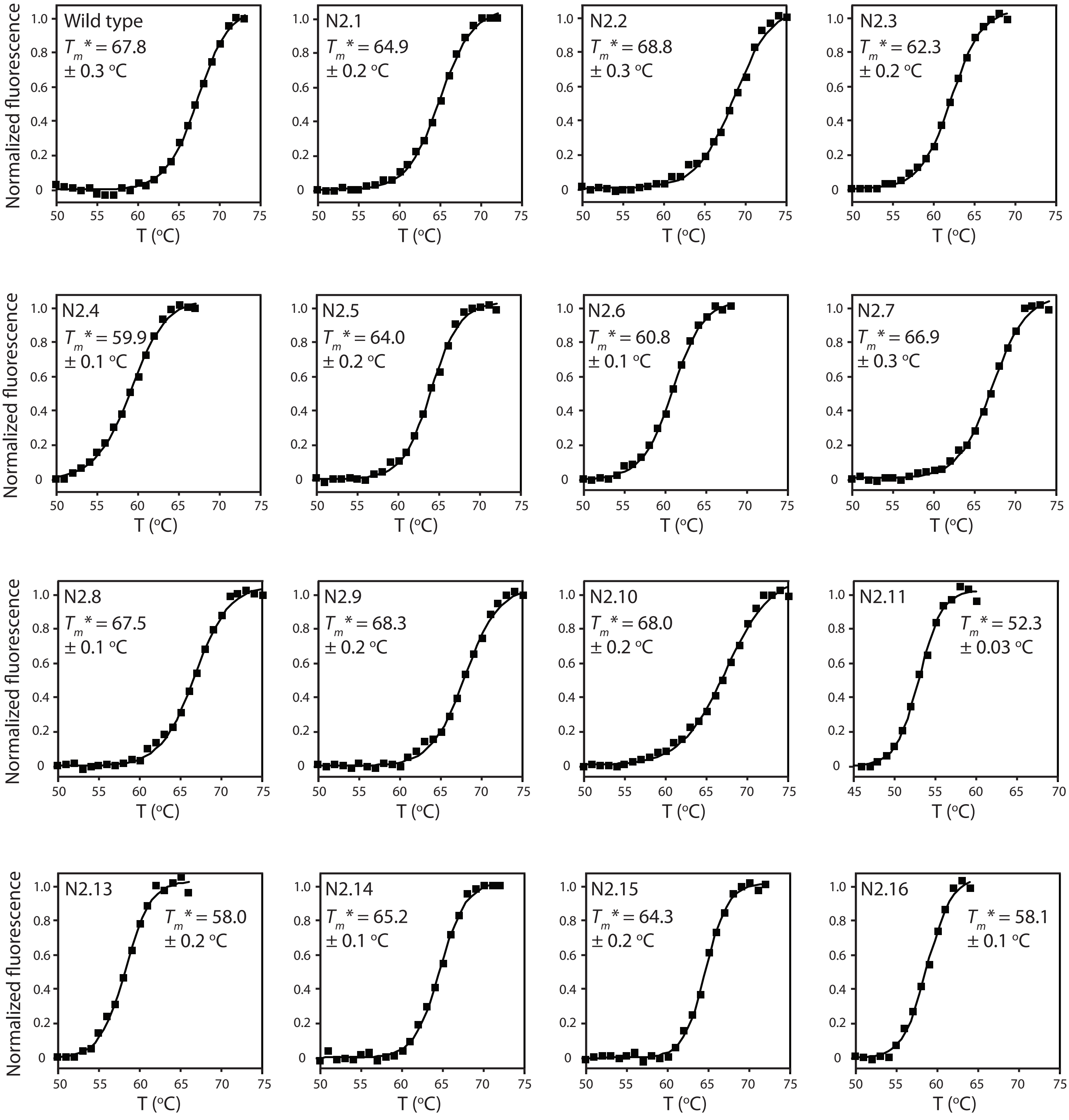

Figure S10. Summary of stability measurements for the wild type and affinity-matured V_HH variants. Representative melt curves are shown for extrinsic fluorescence measurements of V_HH unfolding as a function of temperature. The stability analysis was performed as described in Figure 5. The apparent melting temperature (T_m*) values are averages for three independent experiments, and the errors are standard deviations.

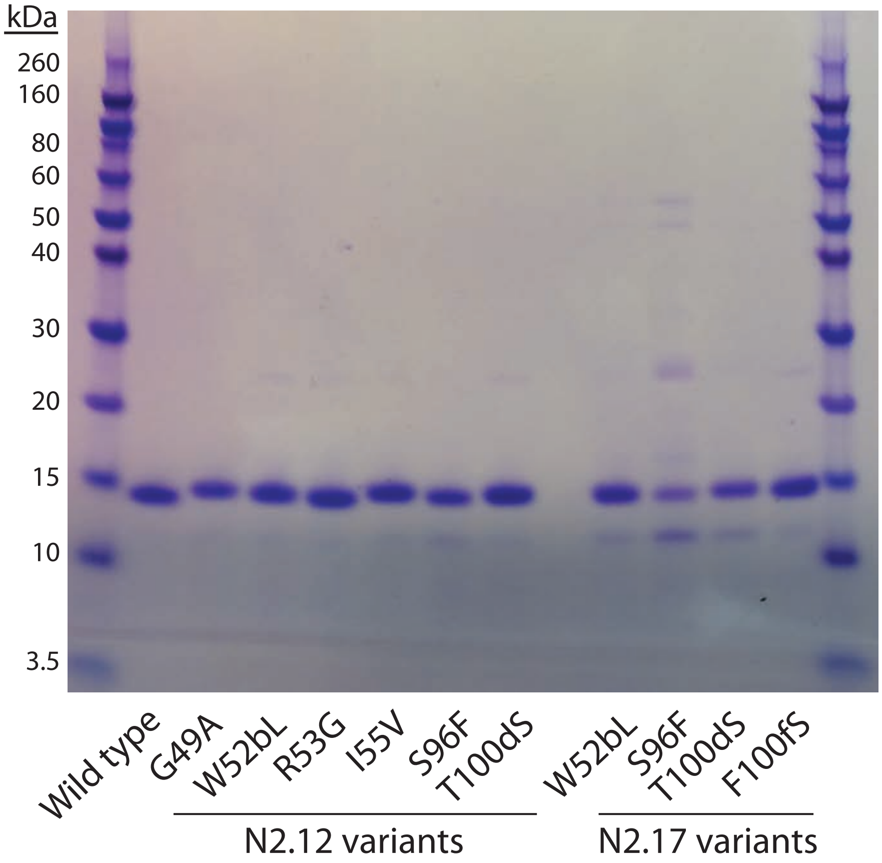

Figure S11. SDS-PAGE analysis of wild type and reversion mutants of affinity-matured V_HH antibodies. The analysis was performed as described in Figure S1.

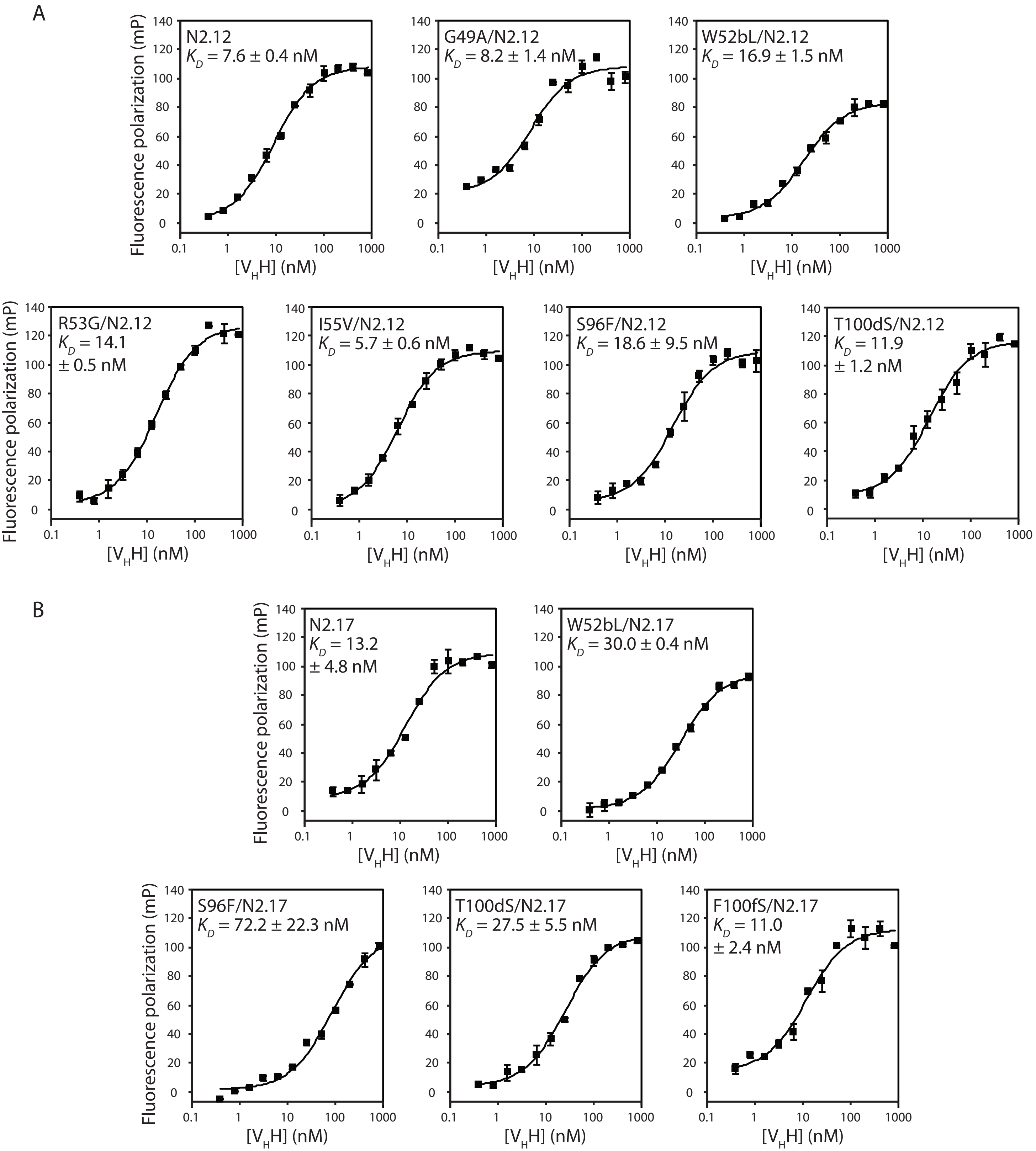

Figure S12. Summary of affinity measurements for reversion mutants of affinity-matured V_HH antibodies. Representative binding curves for measuring V_HH affinity using fluorescence polarization are shown for single reversion mutations of the (A) N2.12 and (B) N2.17 V_HH domains. The affinity analysis was performed as described in Figure 5. The equilibrium dissociation constant (K_D) values are averages for three independent experiments, and the errors are standard deviations.

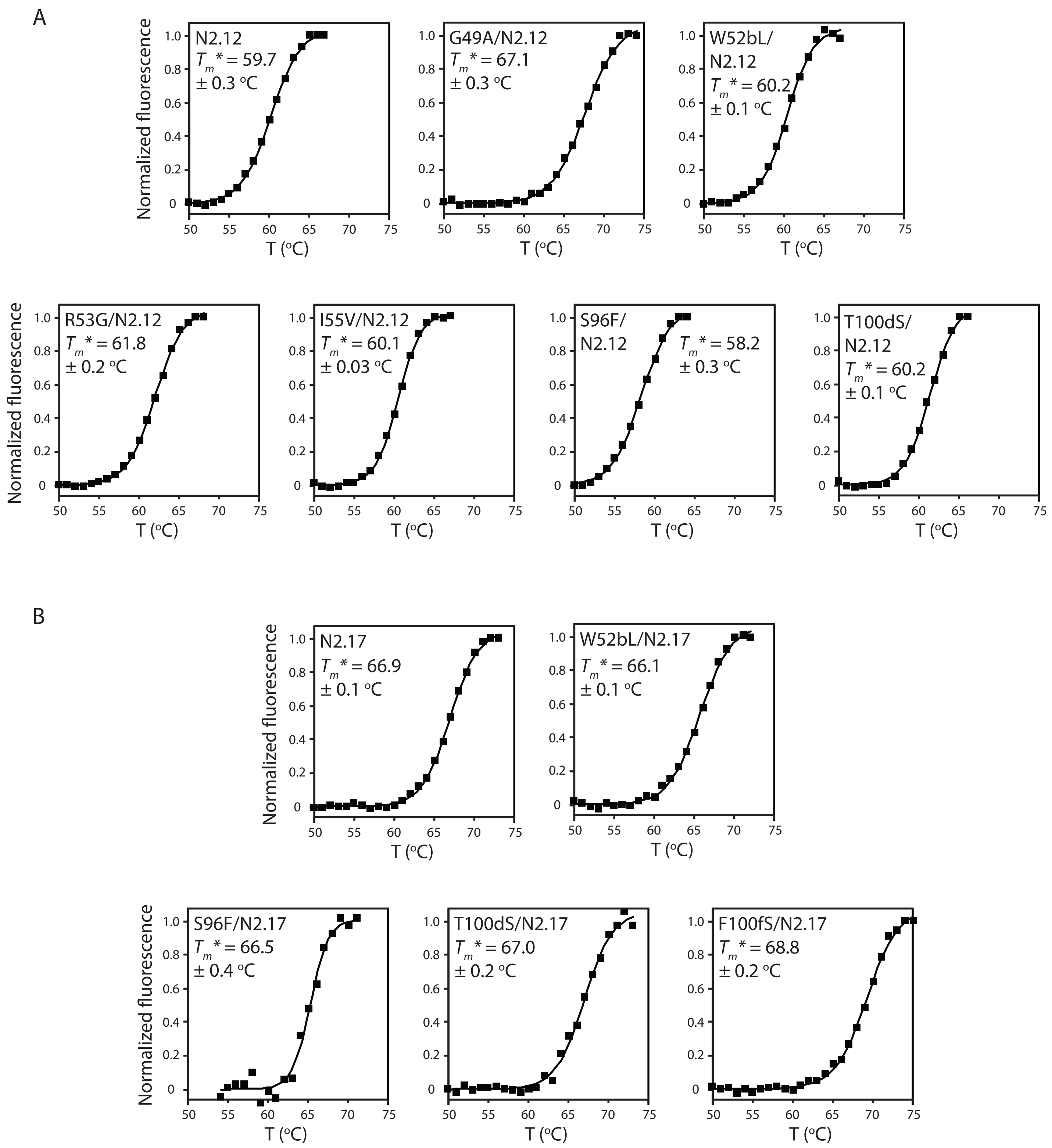

Figure S13. Summary of stability measurements for reversion mutants of affinity-matured V_HH antibodies. Representative melt curves for V_HH unfolding as a function of temperature are shown for single reversion mutations of the (A) N2.12 and (B) N2.17 V_HH domains. The stability analysis was performed as described in Figure 5. The apparent melting temperature (T_m*) values are averages for three independent experiments, and the errors are standard deviations.
